# Application of Intranasal Administration in the Delivery of Antidepressant Active Ingredients

**DOI:** 10.3390/pharmaceutics14102070

**Published:** 2022-09-28

**Authors:** Zhiyu Jin, Yu Han, Danshen Zhang, Zhongqiu Li, Yongshuai Jing, Beibei Hu, Shiguo Sun

**Affiliations:** College of Chemistry and Pharmaceutical Engineering, Hebei University of Science and Technology, Shijiazhuang 050018, China

**Keywords:** depression, blood–brain barrier, brain targeting, antidepressant active ingredients, intranasal administration, challenges of delivery

## Abstract

As a mental disease in modern society, depression shows an increasing occurrence, with low cure rate and high recurrence rate. It has become the most disabling disease in the world. At present, the treatment of depression is mainly based on drug therapy combined with psychological therapy, physical therapy, and other adjuvant therapy methods. Antidepressants are primarily administered peripherally (oral and intravenous) and have a slow onset of action. Antidepressant active ingredients, such as neuropeptides, natural active ingredients, and some chemical agents, are limited by factors such as the blood–brain barrier (BBB), first-pass metabolism, and extensive adverse effects caused by systemic administration. The potential anatomical link between the non-invasive nose–brain pathway and the lesion site of depression may provide a more attractive option for the delivery of antidepressant active ingredients. The purpose of this article is to describe the specific link between intranasal administration and depression, the challenges of intranasal administration, as well as studies of intranasal administration of antidepressant active ingredients.

## 1. Introduction

Depression is a very common central nervous system (CNS) disorder disease worldwide. Clinical symptoms include chronic depression, apathy, loss of appetite, and loss of interest. According to the World Health Organization, more than 350 million people worldwide suffer from depression, hundreds of thousands commit suicide each year, and the number is rising rapidly. More than 75% of patients in countries with a shortage of medical resources and healthcare personnel go untreated [1]. The clinical treatment of depression is still characterized by a low cure rate, high recurrence rate, residual symptoms, dysfunction, and high risk of self-injury and suicide, which imposes a serious burden on physical and mental health and economic levels around the world [2,3]. After decades of research, the most widely accepted treatment strategy for depression is a combination of medication, psychotherapy, and physical therapy. Due to the social environment, psychological burden, and other reasons, the use of antidepressants is generally accepted by patients. There are several antidepressants on the market, most of which are administered orally in tablets or capsules (Selegiline in the transdermal patch, Esketamine in the nasal spray, and Brexanolone in the intravenous infusion). Antidepressants are absorbed by different untargeted tissues and organs in the gastrointestinal tract or after absorption into the blood circulatory system, resulting in systemic clearance (in oral and parenteral route) and widespread adverse reactions such as drowsiness, weight gain, constipation, dry mouth, and dizziness [4,5,6,7]. Invasive drug administration of the brain can cause great discomfort to patients. Some depressive patients have marked relief or disappearance of depressive symptoms after taking antidepressants for some time. However, there is also a subset of patients (major depressive disorder, MDD) who do not respond to two or more first-line antidepressants and have acute or severe suicidal thoughts. Drug therapy is limited because of the complexity of the pathogenesis of depression and the variability of individual patients [8]. The current clinical situation is that depressive symptoms are relieved after 2 to 3 weeks or even longer after oral first-line antidepressants. The early stage after taking antidepressants is also the stage where adverse reactions are prominent. During this incubation period, patients are at increased risk of disability or suicide (especially in patients ≤ 24 years of age), aggravated disease, and decreased medication compliance. Therefore, there is an urgent need to find new antidepressant active ingredients and routes of administration with a fast onset of action and fewer side effects.

**Figure 1:**
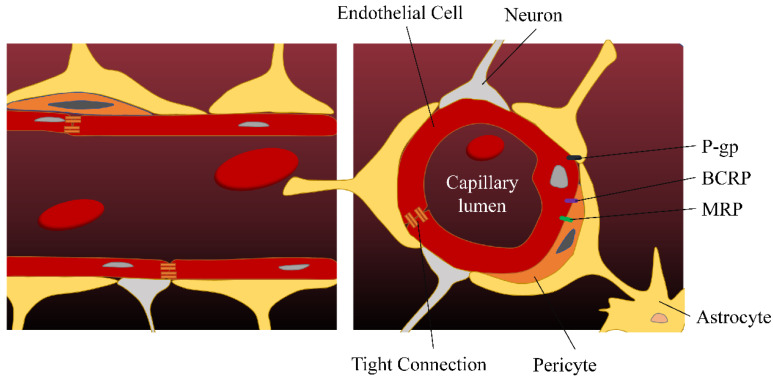
The blood–brain barrier. P-gp, P-glycoprotein; BCRP, breast cancer resistance protein; MRP, multidrug resistance-associated protein.

Due to BBB, extensive metabolism, high protein binding rate, and systemic side effects, many ingredients with antidepressant activity cannot exert effective therapeutic effects by oral or injection administration. The BBB is mainly composed of microvascular endothelial cells, mural cells, and glial cell astrocytes (Figure 1). In contrast to inner cells in other tissues, brain endothelial cells are connected by tight junctions (TJs) and have very little vesicle-mediated transcellular transport. The endothelium of the brain contains a variety of enzymes that inactivate certain neurotransmitters, antidepressants, and toxins, preventing them from entering the brain. In addition, efflux transporters, which are located on the blood side of the endothelial cells, use energy to transport passively diffused lipophilic molecules back into the blood, especially the P-glycoprotein (P-gp) [9,10,11]. Preclinical studies have shown that many antidepressants are substrates of P-gp, which may affect the distribution of antidepressants to their target of action [12,13]. The effects of ABCB1 polymorphisms on P-gp expression and antidepressant transport were significantly different between individuals, which may lead to treatment variability [14].

Some first-line antidepressants, off-label drugs, peptides, natural active ingredients, and other substances with antidepressant activity are limited by the choice of route of administration because of their properties. Even when administered intravenously, some drugs still enter the liver for degradation and are restricted by the BBB. Intracerebral or spinal injections are the most direct methods of delivering drugs to the brain but are invasive and cause great discomfort to patients. Invasive drug delivery is not suitable for depression, which requires long-term treatment. Subsequent increasing studies have shown that intranasal administration can treat brain diseases through the bypassing of BBB, olfactory nerve pathways, trigeminal nerve pathway, and mucosal epithelial pathways, to get the drugs to the CNS [15,16,17,18]. Compared with traditional antidepressant administration routes (oral administration, intravenous administration, intramuscular injection, etc.), intranasal has the advantages of avoiding first-pass effect, improving bioavailability, short onset time, small dose of administration, less toxic and adverse reactions on the body, and good patient compliance [17]. The direct non-invasive pathway between the nose and the brain is undoubtedly one of the best options for the limited antidepressant active ingredients to enter the CNS to exert antidepressant effects. The purpose of this review is to present the unique anatomical and physiological link between the nose–brain pathway and the lesion site of depression. For example, the olfactory system has a high degree of overlap with areas that process emotions and memory functions [19,20]. Furthermore, this review also summarizes studies of antidepressant active ingredients (off-label drugs, peptides and natural active ingredients, etc.), in addition to first-line antidepressants and the delivery carriers in intranasal administration.

## 2. Unique Advantages of Intranasal Administration in the Treatment of Depression

The nose is the starting part of the respiratory pathway and olfactory organs. The nose is divided into two different compartments by the nasal septum. The surface area of the human nasal mucosa is 150 cm^2^~160 cm^2^, the thickness of nasal mucosa is about 2~5 mm, and the average pH value of nasal mucus is 5.5~6.5 [18,21]. The nasal cavity can be divided into three areas according to their structure and function: the nasal vestibule, olfactory region, and respiratory region.

### 2.1. Direct Pathway

**Figure 2:**
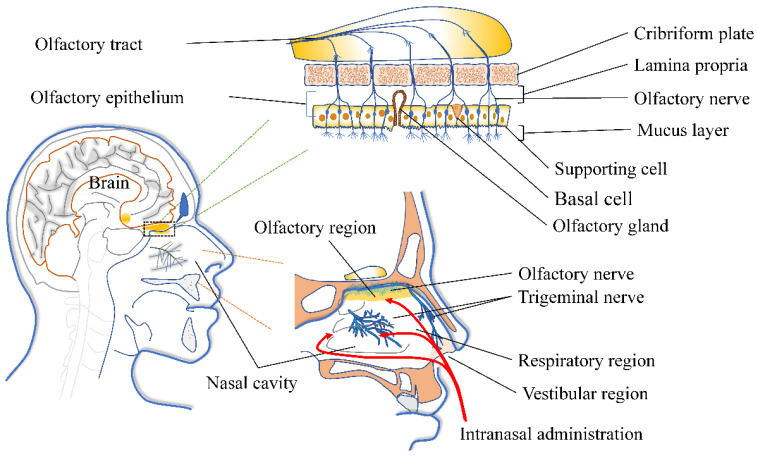
Anatomical schematic of the non-invasive pathway between nose and brain. Adapted with permission from Ref. [24]. Copyright 2018, Elsevier.

The nasal cavity is the only non-invasive direct route between the CNS and the external environment. The nasal vestibular area is located in the front of the nasal cavity with a very small surface area, only about 0.6 cm^2^. Parts of the nasal vestibular area are skin tissue. There are nasal hairs and mucus that block and filter particulate matter; so, the absorption capacity of antidepressants is limited in the nasal vestibule [22]. The olfactory area is distributed on the medial surface of the superior turbinate, part of the middle turbinate, and the corresponding part of the septum [8]. The surface area is relatively small, only about 10 cm^2^. The cells of the olfactory region are composed of olfactory cells, supporting cells, basal cells, and trigeminal nerve cells. The lamina propria (LP)—the relatively loose connective tissue layer beneath the epithelial cells—contains nerves, blood vessels, and lymphatic vessels. Olfactory sensory neurons (OSNs) are bipolar neurons. Axons from OSNs expressing the same odorant receptors aggregate in LP to form axon bundles that pass through the ethmoid plate and terminate in glomeruli formed within the olfactory bulb (Figure 2) [23].

The glomerulus of the olfactory bulb is the only transit point between the peripheral and central olfactory systems. OSNs synapse with mitral cells and tuft cells to project secondary and tertiary olfactory structures. Olfactory information is transmitted to secondary olfactory structures, particularly the piriform cortex and amygdala cortex. Tertiary olfactory structures include the thalamus, hypothalamus, amygdala, hippocampus, orbitofrontal cortex, and insular cortex. These regions are closely associated with the expression of mood and memory function in depression [19,20]. Depressed patients are often associated with reduced olfactory bulb volume and olfactory dysfunction, and people with olfactory dysfunction are at higher risk for depression [25,26]. The causal relationship between the olfactory system and depression is unclear, but the correlation between the severity of olfactory disturbance and the severity of depression have been established. Melatonin MT_1_ and MT_2_ receptors are expressed in the glomerular layer of the olfactory bulb and coupled to Gi protein. Studies have shown that modulation of melatonin receptors expressed in the olfactory bulb can ameliorate 6-hydroxydopamine-induced depression-like behaviors [27,28]. By intranasal administration, the olfactory bulb can serve as a target for the antidepressant effects of melatonin and melatonin analogs.

Olfactory nerve cilia, located on the surface of the olfactory mucosa, can internalize antidepressants, which are then slowly transported in the axoplasm to synapses within the olfactory bulb that connect with secondary olfactory neurons. Afterward, antidepressants are delivered to the prefrontal cortex, hippocampus, and other parts along the olfactory conduction pathway, possibly by repeating this process. Antidepressants need to be transported through the axons of neurons in the olfactory system at least through the tertiary neurons to reach the hippocampus. There are complex possibilities for interneuronal transfer and axoplasmic transport. Further studies are needed on drug delivery in the perineuronal and intraneuronal spaces and transfer between neurons. 

The axons of olfactory neurons are surrounded by a series of olfactory ensheathing cells, and their outer layers are covered by additional neuro fibroblasts (ONFs). The ONFs layer is continuous to the meninges that cover the brain, which means that the perineural space formed between the olfactory sheath cell layer and the neuro fibroblasts are continuous to the subarachnoid space [29]. Antidepressants can enter LP through transcellular or paracellular pathways of the olfactory epithelium, and then along the perineural space into the olfactory bulb and cerebrospinal fluid [30]. Antidepressants need to pass through TJs to reach LP, where OSNs undergo apoptosis and replacement in about 30 to 60 days to protect the brain from airborne contaminants, bacteria, and so on. During this period, a potential delay between lysis and regrowth of OSNs leaves gaps between the tightly connected nasal epithelial cells, resulting in increased permeability [29,31]. In addition, increased permeability of drug absorption is associated with phosphorylation of closed-protein, such as protein kinase signaling or certain substances [32,33]. The characteristic of this pathway is that its effect is faster than that of the olfactory nerve pathway. As the olfactory bulb is located below and anterior to the orbital surface of the frontal lobe of the cerebral hemisphere, antidepressants can be rapidly distributed to the close prefrontal cortex.

The trigeminal nerve is another nerve pathway that connects the nose to the brain, innervating the olfactory and respiratory mucosa [7,29,34]. The trigeminal nerve originates from the pons, where the ocular and maxillary branches supply the nasal cavity. Unlike the axonal bundles of the OSNs, the trigeminal nerve is mainly composed of the myelin sheath made of Schwann cells. The non-myelinated branches of the trigeminal nerve supply olfactory mucosal vessels and regulate blood flow. Trigeminal nerve endings (and their associated arteries) are located below LP and nasal mucosa TJs and do not penetrate the epithelial surface like OSNs [35,36]. Insulin, which has antidepressant activity, was administered intranasally, and fluorescently labeled insulin was found to reach the brain through the extracellular space around the trigeminal nerve [37]. Drugs entering LP may also reach other brain areas, such as the brainstem, through the trigeminal branch [35]. 

The locus coeruleus and raphe nuclei located in the brainstem are the major sites for the synthesis of norepinephrine (NE) and serotonin (5-HT) in the brain. The locus coeruleus and raphe nuclei have a wide range of neuronal projections and play an important role in regulating neuronal activity in areas such as the prefrontal cortex, amygdala, hippocampus, lateral habenula, and anterior cingulate gyrus related to emotion and memory. Neuronal damage in the locus coeruleus and raphe nucleus is strongly associated with depression, especially MDD. The study found that after intranasal administration of radiolabeled immunoglobulin ([^125^I]-IgG), the highest signal in the olfactory bulb and brain stem was observed by radiography [38]. Pang et al. evaluated the pharmacokinetics of intranasal insulin in the rat brain and showed that the highest levels were in the brainstem of the brain, followed by the olfactory bulb, cerebellum, hippocampus, hypothalamus, and striatum [39]. Exogenous active ingredients may be directly distributed to the brainstem through the trigeminal nerve and its surrounding space to repair damaged neurons.

According to previous studies, the convection in the brain perivascular space driven by arterial pulsation is thought to be the main reason for the rapid and widespread distribution of antidepressant active ingredients after entering the brain from the nasal cavity [40,41]. As the olfactory bulb is located below and anterior to the orbital surface of the frontal lobe, antidepressants can be rapidly distributed to the adjacent prefrontal cortex through the olfactory bulb. The cerebrospinal fluid and the subarachnoid space have many vascular punctures deep into the brain, separated from the brain parenchyma by the pia mater. The tiny spaces between these blood vessel punctures and the pia mater allow for the circulation of cerebrospinal fluid, known as the cerebrospinal fluid microcirculation. Antidepressants are better distributed in the brain through perineural, perivascular, and cerebrospinal fluid flow.

### 2.2. Indirect Pathway

The olfactory area, about 130 square centimeters, acts to warm and humidify the inhaled air as well as filter particles and pathogenic microorganisms. The mucosa is the stratified or pseudostratified columnar ciliated epithelium, and the cilia mainly move from the front to the back of the nasopharynx. The mucosa is rich in secretory glands and goblet cells, producing a large number of secretions. The mucosal surface is covered with a layer of mucus blanket, which moves backward with the movement of cilia [42]. The abundant capillaries in the respiratory area make this area a nasal cavity, and when administered, antidepressants are absorbed into the system rather than directly into the brain.

The nasal mucosa is in direct contact with the external environment, where many pathogens exist. Nasal-associated lymphoid tissue (NALT) plays a vital role in maintaining the body’s immunity [43]. NALT is located in the LP and submucosal region of the nasal epithelium and connects to the cervical lymph nodes. This is also a potential pathway for the nose-to-brain antidepressant delivery.

Antidepressants can be absorbed by capillaries and lymphatics through the continuous porous endothelium, or enter the blood circulation in the olfactory area through the LP, avoiding first-pass metabolism [44]. Small lipophilic molecules pass more easily. However, the indirectly transported substances still need to pass through the BBB to enter the CNS or brain tissue; so, the substances that enter the brain through the circulation are usually small molecular weight and strong lipophilic substances [45,46].

From the perspective of physiological anatomy and pathological correlation, intranasal administration may have a more direct antidepressant effect than other routes of administration. Results from different studies suggest that intranasal drug delivery may be through a single route or a combination of different routes. Compared with the traditional administration route, intranasal administration can effectively avoid the extensive metabolism and low permeability of the blood–brain barrier caused by the gastrointestinal route. but also avoid the antidepressants in the circulation of the blood as a result of higher plasma protein binding and distribution in the other route, targeting the organization and improve the concentration of antidepressants in the CNS [2,47]. Non-invasive intranasal administration can effectively shorten the onset time and improve compliance in patients with depression requiring long-term treatment [2,48].

## 3. Challenges of Intranasal Administration

Intranasal administration offers an excellent strategy to overcome the challenge of the complex pathophysiology of brain diseases and drug penetration into the brain. However, the physiological characteristics of the nose, the physicochemical properties of antidepressant active ingredients, and even intranasal drug delivery devices can influence the nose-to-brain delivery of antidepressants [18,49]. 

TJs of the olfactory and respiratory epithelium and their protective mucus lining act as selective filters, reducing antidepressants diffusion and permeability [50]. Although most lipophilic compounds are more permeable to the nasal mucosa, peptides, macromolecules, and small hydrophilic molecule compounds are generally less permeable [34,51]. Permeation enhancers have been shown to improve the absorption of high molecular weight antidepressants by facilitating the production of hydrophilic pores, and increasing membrane fluidity and permeability of TJs [34,52]. Common Permeation enhancers include cyclodextrin, chitosan (CN), surfactant, Cremophor RH40, saponins, etc. [51,53,54]. Gavini E. et al. prepared solid microparticles based on CN or methyl-beta-cyclodextrin to enhance the nose-to-brain delivery of deferoxamine mesylate [55]. Cell-penetrating peptides and penetration accelerating sequences significantly facilitate the delivery of polypeptide antidepressants, such as insulin [56]. Permeation enhancers may cause irritation and toxicity to the nasal mucosa while increasing the permeability of the compounds. The selection of materials and safety experiments is the premise to ensure the safety and effectiveness of prescription, especially for depression requiring long-term administration. Nasal mucus is mainly composed of 90~95% mucin and 2~3% water and tends to dissolve hydrophilic substances [57]. Mucin fibers are usually composed of a proline–threonine–serine backbone with intermitting cysteine-rich domains. Different amino acids in the PTS region are highly glycosylated via O-linked bonding. The degree of mucin glycosylation affects the permeability and viscosity of the mucus [58,59]. There are many cilia on the surface of the olfactory and respiratory areas, which block particles from the external environment from entering the nasal cavity. The olfactory region cilia do not move, and the respiratory region cilia generally toward the pharynx at an average velocity of 5~8 mm per minute; so, the nasal administration of drug particles tends to be cleared in an average time of 20 to 30 min [60]. Inhibitory substances and mucoadhesive materials such as hyaluronan, poloxamer, carbopol, gellan gum, polycarbophil, and other polymers can effectively delay mucociliary clearance and increase the retention time of antidepressants in the nasal cavity, thus increasing drug intake in the brain by interacting with mucin or reversibly/irreversibly inhibiting cilia movement [51]. 

Although there are fewer enzymes in the nasal cavity, they still may affect antidepressant absorption, such as cytochrome P-450 enzyme system, glutathione S-transferase, proteolytic enzyme, and other “Pseudo-first-pass effect” on foreign substances. Enzyme inhibitors or some surfactants such as bestatin, amastatin, boroleucine, fusidic acids, and phospholipids can be used to avoid the metabolism of the compound, improve stability, and thus increase the absorption of the original drug [34]. 

Like the BBB, P-gp act as multidrug resistance pumps, expressed in nasal epithelial cells, and P-gp effusion restricts the entry of most substances into the brain. This effect can be modulated by the use of P-gp inhibitors [61]. For example, cyclosporine A and rifampin as P-gp inhibitors improve the permeability of verapamil after intranasal administration [62].

As the breathing area is much larger than the olfactory area, some of the antidepressants that are administered intranasally will still enter the brain via indirect routes. Therefore, studies have shown that the use of direct access can be increased by combining vasoconstrictors to reduce the absorption of antidepressants through the nasal vessels and to increase the retention time of antidepressants in the nasal mucosa [51,63]. Dhuria S. V. et al. administered an intranasal combination of phenylephrine and neuropeptides and measured the concentration of the drug in CNS tissues and blood. The addition of 1% phenyl epinephrine significantly reduced the absorption of HC and D-KTP in the blood and increased the delivery volume of the olfactory bulb [64]. Vasoconstrictors reduce the peripheral side effects of CNS antidepressants by restricting the absorption of nasal blood vessels into systemic circulation.

In addition to the physiological factors mentioned above, the physicochemical properties and formulation composition of antidepressant active ingredients also have an effect on the nose-to-brain delivery. The molecular weight of the drug is inversely proportional to the percentage absorbed. Lipophilic ingredients are more readily absorbed than hydrophilic ones. Lipophilic small molecule ingredients with a molecular weight of less than 1 kDa can be transported quickly. The absorption of ingredients of less than 300 Da was almost unaffected by molecular weight, while the absorption of antidepressants with molecular weight between 300 Da and 1 kDa was inversely correlated with molecular weight [65]. Currently, the most widely used and marketed antidepressants, such as Escitalopram, Fluoxetine, Duloxetine, and Venlafaxine, have molecular weights ranging from 200 to 400 Da [66,67]. The pH value of the formulation not only ensures the stability of the drug itself but also ensures the stability of the physiological conditions of the nasal cavity. The nasal pH range is 5.0~6.8, and intranasal administration should be close to this to avoid irritation of the nasal mucosa. The drug is always absorbed in a non-ionized state; so, the pKa of the drug should also be taken into account in determining the pH of the formulation [68]. However, changes in temperature, humidity, and some pathological conditions can cause changes in nasal pH [69]. In addition, the prescribed osmotic pressure should also be adapted to the physiological conditions of the nasal cavity; otherwise, it will affect the normal nasal mucosal cell morphology and ciliary movement and further affect the absorption of antidepressants [31,70].

The choice of drug delivery equipment also plays a very important role in whether the drug formulation can be utilized to the maximum extent and play its due therapeutic effect [34,49,71]. Treatment of CNS diseases such as depression requires antidepressants to be delivered to the olfactory region. Traditional pump sprays usually deposit antidepressants in the anterior nasal cavity, which are removed quickly by clearance of the nasal mucosa. The droppers may deposit the olfactory area above the nasal cavity better than nasal pump sprays, but this often requires the patient to lie on his or her back in a head-down, forward–forward position and even professional administration techniques, which can be very inconvenient [72]. Vianase™ device is an electronic atomizer developed by Kurve Technology^®^ that consists of an atomizer and a vortex chamber [73]. The atomizer causes the preparation to produce atomized particles, which, under the action of the vortex chamber, form a vortex, and are ejected from the equipment in this form. Vianase™ device can precisely maximize the delivery of drug to the olfactory region through electronic control. Opt-Poeder device is a bidirectional delivery device that uses the patient’s exhalation as the power to deliver the drug to the targeted site. The device is designed so that when a person exhales, the soft palate closes, preventing antidepressants from entering the respiratory tract and depositing in the nose. The Precision Olfactory Delivery device, designed and manufactured by Impl Neuropharma, is a single-dose delivery device that utilizes inert gas as an impeller to deliver drug to the superior nasal cavity [34,74]. Experimental data indicate that 45% of the dose of the Precision Olfactory Delivery device can be deposited in the upper nasal cavity compared with the traditional pump [75]. Other devices such as the Aero Pump System are also well used in intranasal delivery. However, the proportion of drug deposition in the olfactory region is still relatively low due to these devices; thus, the research and development of more convenient and efficient devices is one of the focuses of current research.

## 4. The Delivery Carriers and Nanocarriers for Intranasal Administration

### 4.1. Polymer-Based Carriers

**Figure 3:**
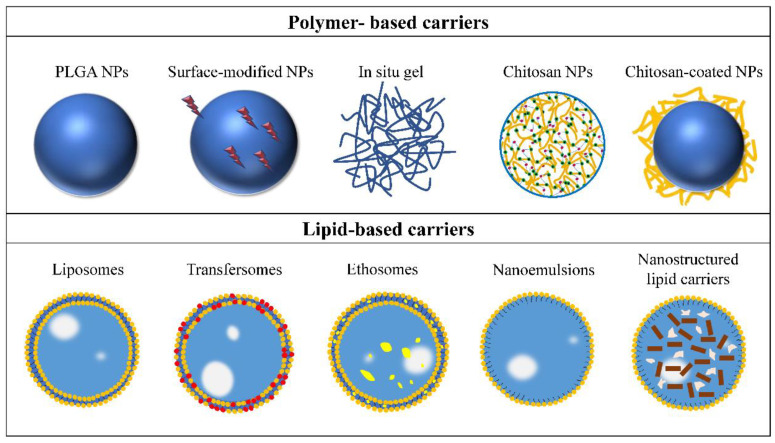
Carriers for intranasal administration of antidepressant active ingredients. Adapted with permission from Ref. [24]. Copyright 2018, Elsevier.

The physiological characteristics of the nose–brain pathway and the physicochemical properties of ingredients lead to a certain degree of limitation for intranasal administration [58,59]. So far, many scholars have conducted studies on the nose-to-brain delivery system to improve the safety and effectiveness of ingredients, alongside the development in polymer technology and pharmaceutical technology. The delivery carriers and nanocarriers have made significant contributions to protecting antidepressants from protein degradation, enhancing olfactory mucosal uptake and CNS utilization and prolonging half-life (Figure 3).

In situ gel refers to a kind of preparation that is transformed from liquid to non-chemical cross-linked semi-solid gel immediately after administration in solution state due to physiological conditions of the administration site or some stimulus factors in the external environment (pH, temperature, ionic strength, etc.) [76,77]. Compared with the traditional drug delivery system, in situ gels prepared with a variety of different polymers or containing different stimulus-induced release factors show good biocompatibility when exposed to the site of administration for a longer time to prolong the retention time of formulation and improve the bioavailability of antidepressants [78,79,80]. The combination of nanocarriers with in situ gels is also a promising strategy.

Poly (lactic-co-glycolic acid) (PLGA), which is approved for therapeutic applications by the U.S. Food and Drug Administration (FDA), is considered one of the most promising synthesized polymers as a drug delivery system due to its biodegradability, biocompatibility, controllable properties, well-defined formulation techniques, and great potential for targeting [31,81,82]. PLGA is a polymer synthesized by the interaction of glycolic acid and lactic acid monomer. 

Chitosan nanoparticles (CN-NPs) not only can open TJs between cells transiently but also reduce the mucociliary clearance, prolonging the retention time of the compound as a biological adhesive material, which enhances the delivery of antidepressants [83,84]. CN is a natural linear polysaccharide cationic and hydrophilic polymer by alkaline hydrolysis of chitin, which is the second most abundant biopolymer in nature. It is an important component of the shells of many lower animals, especially arthropods such as shrimp, crabs, and insects. It also exists in the cell walls of lower plants such as bacteria, algae, and fungi. CN consists of randomly distributed β-(1,4)-linked d-glucosamine (deacetylated) and N-acetyl-d-glucosamine (acetylated) units. Due to its properties of adhesivity, biocompatibility, and biodegradability, CN has been well used in the field of medical engineering [85]. 

Alginate nanoparticles have also been studied and reported by many researchers in nose-to-brain drug delivery [86,87]. Alginate is extracted from brown Marine algae and then processed several times before it can be used as a polymer in pharmaceutical preparations. Alginates are mainly composed of β-D-mannuronic acid and α-L-guluronic acid, and have properties such as biocompatibility, biodegradability, low toxicity, and pH sensitivity [88,89]. Alginate contains a large number of carboxyl groups and is a hydrophilic anionic polymer, showing a certain adhesion. Divalent cations, such as Ca^2+^, exchange ions with cations on α-L-guluronic to form a cross-linked network structure, thus forming alginate hydrogels [90].

Nanoemulsion (NE) is a liquid nano-dispersion system formed by two kinds of insoluble liquid stabilized by surfactant and co-surfactant (O/W; W/O). NEs have been widely used in nasal and brain delivery of insoluble antidepressants due to excellent solubility, thermodynamic stability, and easy preparation [91,92,93]. CN is often added to NEs to reduce clarity and prolong the retention time of antidepressants in the nasal cavity [91,94].

### 4.2. Lipid-Based Carriers

Compared with polymer carriers, lipid carriers have better biocompatibility than synthetic polymers because the materials of lipid carriers are basically derived from natural materials [95]. The degradation of polymer carriers in vivo is often accompanied by an increase in toxicity, while the degradation products of lipid carriers have low immunogenicity. Lipid carriers can effectively avoid being cleared by the immune system in the body and achieve long cycles. Liposome is a kind of structure similar to the cell membrane, mainly composed of phospholipids’ double-layer synthetic membrane. In water-soluble solvents, the hydrophobic tails are clustered close to each other inside the bilayer phospholipids, while the hydrophilic heads are exposed outward to the aqueous phase, forming vesicles with bilayer molecular structure [96]. Liposomes can effectively protect the stability of encapsulated antidepressants, reduce drug toxicity, and play a slow release, prolonging the action time of antidepressants [97]. In addition, some studies have used altered phospholipid vesicles, such as transferosomes, ethosomes, and phospholipid magnesomes, for intranasal administration. Adding glycerol and ethanol makes the phospholipid vesicles softer, enhancing the permeability to the nose–brain pathway [98,99,100].

It is noteworthy that due to the traditional lipid carrier containing unsaturated chains, it is easy to fuse, oxidize, and hydrolyze as a solution form; so, the half-life of classic liposomes is short and the stability is poor. In order to overcome the defects of liposome preparations, new lipid-based forms for drug delivery have been gradually developed. For instance, solid lipid nanoparticles (SLNs) and nanostructured lipid carriers (NLCs) have been widely used in drug delivery [101,102]. SLNs are nanoparticles made using one or more lipids—such as triglycerides, lecithin, fatty acids, etc.—as carrier materials and combined with surfactants as stabilizers to form solid nanoparticles at body temperature and room temperature. Compared with traditional liposomes, SLNs have lower cytotoxicity, higher stability, and bioavailability and can more effectively maintain drug stability and control drug release, as well as mature the industrial production process [96,101,102,103,104]. Although SLNs have many advantages as nanoscale drug carriers, its disadvantages should not be ignored. For example, SLNs still have the problem of gelation in the dispersed phase and expulsion of encapsulated antidepressants resulting from β-modification during storage, and low encapsulation rates and drug loads due to the fact that cavities are not allowed to occur within the lipoid nucleus during crystallization [104]. NLCs are the second generation of lipid nanomaterials. Different from SLNs, NLCs are composed of liquid lipids and solid lipids combined with surfactants as stabilizer to form nanoparticles that are also solid at room temperature and body temperature. NLCs significantly enhance drug loading due to the existence of liquid lipids, imperfect crystal order and loose structure of nanoparticles, and reduced drug leakage caused by β-modification during storage. The combined use of different carriers, or the functional modification of classical carriers, has aroused extensive interest in facilitating intranasal drug delivery and has promising prospects.

## 5. Intranasal Administration of Antidepressant Active Ingredients

### 5.1. Antidepressants

**Figure 4:**
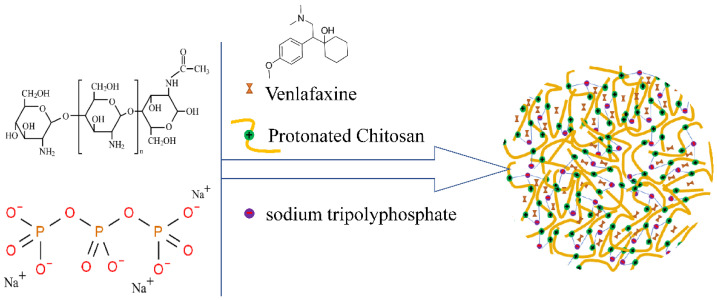
Chitosan is protonated under acidic conditions, and then ionically cross-linked with negatively charged sodium tripolyphosphate to prepare chitosan nanoparticles.

Venlafaxine (VLF) inhibits central 5-HT and NE neuronal reuptake, thereby increasing levels in the synaptic cleft between neurons in the brain. Oral VLF has low bioavailability, a short half-life, delayed onset of action, and significant systemic adverse reactions [105,106]. Cayero-Otero et al. prepared VLF-loaded PLGA-NPs and modified the nanoparticles with transferrin and specific peptide against transferrin receptor. Compared with plain NPs (around −25 mV), the amide reaction of the carboxylic acid terminus on the PLGA surface with the amino group of the peptide resulted in a less negative zeta potential value. The release rate of ordinary nanoparticles is higher than that of modified nanoparticles. Cell viability of h-CMEC/D3 cells is more than 85% in the MTT assay. In vivo biodistribution studies showed higher concentrations of plain fluorescent NPs than functionalized NPs in the brain after 30 min of administration. The authors hypothesize that the reason for this result is that plain NPs enter the brain through direct neural and olfactory epithelial pathways between the nose and brain. However, the speed and degree of functionalized NPs transport, which were mediated by the transferrin receptor expressed by the BBB, are not superior to the direct pathway between nose and brain [107]. Baboota S. et al. published a report about the new preparation of VLF-loaded CN-NPs by using CN and sodium tripolyphosphate ionic gel (Figure 4). CN is positively charged and has mucosal adhesion. It can inversely open TJs between nasal mucosal epithelial cells. The cumulative drug permeability after 24 h in VLF CN-NPs was nearly 3 times compared with VLF solution. VLF CN-NPs showed a more significant antidepressant effect than VLF solution on chronic depression rats by forced swimming method [108]. The properties of CN could be modified by functional groups, such as cross-linking, etherification, carboxymethylation, and graft copolymerization [109]. 

Desvenlafaxine (DVF) is a 5-HT and NE reuptake inhibitor. Systemic side effects were obvious after oral administration of DVF [110]. Tong et al. prepared desgavenlafaxine-loaded PLGA-CN-NPs. Intranasal PLGA-CN-NPs can increase the retention time and permeability of DVF in the nasal mucosa, avoiding degradation in the gastrointestinal tract, and liver intranasal PLGA-CN-NPs can increase the residence time and permeability of DVF in the nasal mucosa, avoiding degradation in the gastrointestinal tract and liver and bypassing BBB. In a rodent model of depression, compared with intranasal DVF solution and oral administration, increased levels of 5-HT and NE in the brain showed a more pronounced antidepressant effect. Pharmacokinetic parameters such as concentration, half-life, and AUC in the brain after intranasal administration were higher than those after intravenous [111].

As an atypical antidepressant, agomelatine has antidepressant effects by agonizing MT1, MT2 and antagonizing 5-HT2C. Agomelatine is well and rapidly absorbed (≥80%) after oral administration. Peak plasma concentrations are reached within 1–2 h after oral administration. However, the first-pass metabolism of agomelatine in the liver is obvious and the protein binding rate in the blood is high. Yasmin et al. prepared an intranasal formulation of agomelatine-loaded NE-thermosensitive in situ gel with CN. Pharmacokinetic study in Wistar rats showed that plasma concentration in the brain was 2.82 times higher than that of the intravenous suspension via the intranasal route [112]. The nasal solid lipid nanoparticles prepared by Ahmed et al. were superior to the oral suspension in brain concentration, AUC_0–360min_, and absolute bioavailability (44.44%) [113]. Their other study combined drug-loaded SLNs-NPs with in situ gels, which also showed that intranasal administration has advantages over the oral route [114]. In the forced swim test, polymer nanoparticles receiving intranasal agomelatine significantly reduced immobility time in mice compared with suspension [115]. 

Duloxetine is a 5-HT and NE reuptake inhibitor. Duloxetine is acid intolerant, and its oral first-pass metabolism significantly affects bioavailability. Its concentration in cerebrospinal fluid is low. Baboota et al. loaded duloxetine into solid/liquid lipid-based nanostructured lipid carriers (DLX-NLCs). Intranasal DLX-NLCs showed higher concentrations in blood and brain compared with DLX solution and oral route, which showed the same results in behavioral tests in mice. Intranasal NLCs were 8-fold higher in brain concentrations than intravenous DLX. The controlled release provides the possibility for the sustained action of DLX in the brain [116,117]. Fares et al. designed a thiomer gel loaded with duloxetine proniosomes to increase the retention time, sustained release, and penetration of DLX in the nasal mucosa (1.86 times that of duloxetine proniosomes) [118]. Shah et al. designed intranasal DLX in situ cubo-gel by a central composite approach. Compared with the intranasal DLX solution, brain bioavailability was increased by 1.96 times [119].

Paroxetine, a phenylpiperidine derivative, selectively suppresses the 5-HT transporter, blocks the reuptake of 5-HT by the presynaptic membrane, and prolongs and increases the effect of 5-HT, thereby producing antidepressant effects. However, paroxetine has extensive first-pass metabolism and the BBB obstruction that limits its access to the brain [120]. Baboota S. et al. successfully developed an O/W type NE loaded with paroxetine for the study of intranasal treatment of depression. The permeability of paroxetine NEs was 2.57 times higher than that of its suspension via permeation studies. Results of behavioral studies in rats showed that intranasal administration of paroxetine NEs significantly improved behavioral activity in depressed rats compared with the oral suspension of paroxetine [121]. In addition, in nose-to-brain delivery for brain diseases, coating with adhesives such as CN is often added to NEs to reduce clarity and prolong the retention time of antidepressants in the nasal cavity [91,94]. Fortuna et al. prepared three nanostructured lipid carriers loaded with escitalopram and paroxetine. In vivo studies showed that intranasal delivery of the drug had similar pharmacokinetic parameters to intravenous administration, whereas escitalopram did not exhibit significant direct nasobrain delivery but reduced exposure elsewhere. In contrast, intranasal delivery of paroxetine-loaded borneol-NLCs significantly increased the concentration in the brain (5-fold), showing good brain targeting [122].

Trazodone is a tetracyclic atypical antidepressant that inhibits 5-HT reuptake and antagonizes 5-HT2 receptors in the presynaptic membrane to promote 5-HT release. Plasma concentrations and onset of oral trazodone are significantly affected by food, and there are significant systemic side effects. Sayyed et al. radiolabeled trazodone and compared the pharmacokinetic parameters of intranasal delivery of ^131^I-TZ solution, ^131^I-TZ microemulsion, and intravenous injection of ^131^I-TZ solution. Intranasal ^131^I-TZ microemulsion had sustained and higher brain uptake at any time tested than the other two formulations and routes. In addition, the blood exposure of intranasal ^131^I-TZ microemulsion was lower than that of intravenous injection, reducing systemic toxicity [123].

Quetiapine fumarate can clinically treat manic episodes of bipolar disorder by antagonizing 5-HT2A receptors and D2 receptors. Oral quetiapine fumarate is also widely metabolized and has poor bioavailability. In addition, quetiapine fumarate is also a substrate of P-gp, and its absorption and brain distribution are limited. The intranasal NE prepared by Boche et al. enabled quetiapine fumarate to have higher brain-targeting efficiency and a shorter time to peak plasma concentration than intravenous injection [124]. In another comparative study, the brain bioavailability of quetiapine fumarate of CN-coated microemulsion was 3.8-fold and 2.7-fold higher than that of drug solution and CN-free microemulsion, respectively [125]. CN has an obvious promoting effect on the nasal mucosa.

Doxepin hydrochloride is a tricyclic antidepressant and anxiolytic. Doxepin hydrochloride has extensive first-pass metabolism and poor oral bioavailability (13–45%). Hema et al. prepared thermoreversible biogels based on CN and glycerophosphate loaded with doxepin hydrochloride. The formulation gelled rapidly at 37 °C, showing good drug permeability and long residence time. Compared with doxepin hydrochloride solution, the thermoreversible biogel showed more advantages in immobility time and swimming activity count in mice after 13 days of drug administration [126].

Intranasal administration is not appropriate for the type of antidepressant used. Some first-line antidepressants, such as escitalopram, have a good oral absorption effect, are not significantly metabolized, have high absolute bioavailability, and have a high ability to cross the BBB. Such antidepressants may be more suitable for oral formulations. Compared with intranasal administration, oral administration is undoubtedly more convenient and more easily accepted by the public who are now taking medication alone.

### 5.2. Off-Label Drugs

**Figure 5:**
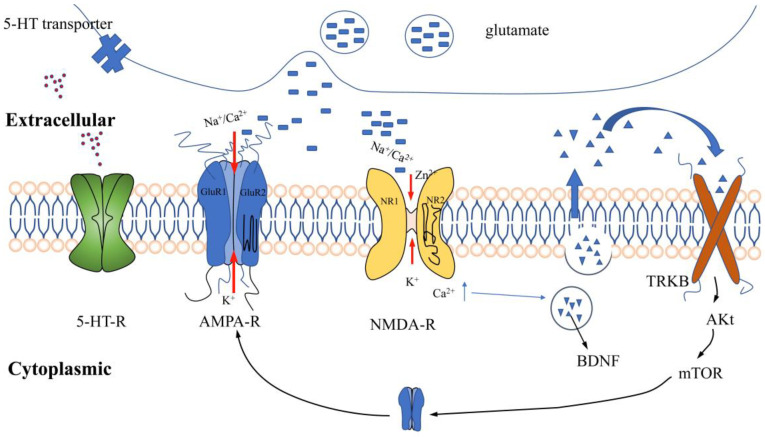
Receptors for antidepressant action.

The rapid and potent antidepressant effect of ketamine for anesthesia is the most striking discovery in recent decades. The U.S. FDA approved the intranasal S-enantiomer of ketamine (Esketamine) in 2019 for the treatment of MDD in adults. The S-enantiomer of ketamine is more potent on NMDAR than the R-enantiomer and racemic ketamine [127,128]. R-enantiomer of ketamine exhibits more potent and longer-lasting antidepressant effects [129]. Studies have shown that in addition to antagonizing N-methyl-D-aspartate (NMDA) receptors, ketamine also inhibits possible mechanisms such as opioid receptors and monoamine transporters to exert antidepressant effects. It is now clear that ketamine, whether administered intravenously or intranasally, has a higher bioavailability than the oral route, and has a more rapid and significant effect than traditional antidepressants with delayed onset of action. Due to the plasma elimination half-life of ketamine of 2–4 h and the discomfort associated with invasive administration, delivery of ketamine directly to the brain via the nasal cavity is a more advantageous strategy (Figure 5).

Studies have shown that dopamine plays an indispensable role in the regulation of neural activity in states such as fear and anxiety. The ability of dopamine to cross the BBB is extremely poor, but intranasal dopamine exhibits antidepressant activity in the forced swim test [130]. Only dihydroxyphenylacetic acid produced by monoamine oxidase metabolism was detected in the CNS following intranasal dopamine delivery. However, the submucosal MAO saturates rapidly after administration, with little effect on the delivered dopamine content [131]. Liu et al. designed an interfering peptide capable of disrupting the interaction between dopamine D1 and D2 receptors. Due to the invasiveness of injection into the brain and cerebrospinal fluid and the restriction of the BBB to the peptide, they chose the route of intranasal administration. Intranasal administration showed similar antidepressant effects to imipramine, and disruptive effects of interfering peptides could be detected in the prefrontal cortex [132].

Amisulpride is a second-generation atypical antipsychotic drug approved by the FDA for schizophrenia. Amisulpride selectively binds to D2/D3 dopaminergic receptors in the limbic system [133]. Multiple reports indicate that amisulpride blocks presynaptic dopamine D2/D3 receptors at low doses to promote dopamine transmission and antagonize 5-HT7 receptors to play an antidepressant role [134,135,136]. The absolute bioavailability of amisulpride after oral absorption is low (48%), and 25% to 50% of intravenous or oral administration will be eliminated with the original drug in the urine. Long-term use can also produce leukopenia or agranulocytosis [137,138]. Kokare et al. prepared amisulpride-loaded lipid-based poloxamer-gellan gum nanoemulgel for nose-to-brain delivery. Pharmacokinetic studies in Wistar rats showed that the intranasal Cmax of the brain was 3.39 times higher than that of the intravenous administration. Further, intranasal administration within one month did not affect blood leukocyte and granulocyte counts [139].

Aripiprazole—also a second-generation atypical antipsychotic; a partial agonist of dopamine D2, D3, and 5-HT1A receptors; and an antagonist of 5-HT2A receptors—has efficacy as a first-line antidepressant enhancer [140,141]. Although aripiprazole is well absorbed after oral administration, it undergoes extensive metabolism. Moreover, aripiprazole and its active metabolite dehydroaripiprazole are substrates for P-gp, limited by BBB permeability, resulting in low CNS bioavailability [142,143]. Hira et al. prepared aripiprazole-loaded mucoadhesive NE (ARP-MNE). Pharmacokinetic studies with single-dose administration showed that the plasma concentration in the brain of intranasal ARP-MNE was 1.44 and 6.03 times higher than that of intranasal and intravenous ARP-NE, respectively, and the Tmax was smaller than that of intravenously administered ARP-NE. Intranasal administration of ARP-MNE had higher values of drug targeting efficiency (96.90%) and drug targeting potential (89.73%) [144]. Aripiprazole-loaded poly(caprolactone) nanoparticles (APNPs) were prepared by Krutika et al. The AUC_0–8h_ of Aripiprazole in the rat brain administered by the intranasal route of APNPs was approximately twice that of the intravenous route [145]. Somayeh et al. developed an ion-sensitive in situ gel containing Aripiprazole-loaded nanotransfersomes (APZ-TFS-Gel) for intranasal administration. Animal behavioral evaluations (swimming and climbing time, locomotor activity, and immobility time) showed that intranasal delivery of APZ-TFS-Gel was more effective for improving depression than oral or other formulation groups [146].

Selegiline is an irreversible type B monoamine oxidase (MAO-B) inhibitor clinically used to treat Parkinson’s disease. High-dose selegiline is an irreversible inhibitor of MAO-A and MAO-B for the treatment of MDD. Selegiline transdermal patch has been clinically used to treat MDD. Selegiline can increase the content of dopamine in the hippocampus, activate D1 receptors, and regulate neuronal plasticity to play an antidepressant effect. However, oral selegiline has high first-pass metabolism and low bioavailability [147]. Wairkar et al. prepared selegiline-loaded CN-NPs and compared the pharmacokinetic parameters of selegiline delivered in the brain and plasma by different formulations and routes of administration. The C_max_ of plain solution of selegiline in the brain and plasma by intranasal administration (T_max_ = 5 min) was 20 and 12 times higher, respectively, compared with oral administration (T_max_ = 15 min). Furthermore, intranasal administration of selegiline-loaded CN-NPs and mucoadhesive thermosensitive gel showed superior formulation advantages compared with the AUC_0–24h_ of plain solution [148,149]. There are also numerous studies on the enhancement of its bioavailability in the brain by intranasal delivery of selegiline-loaded vehicles, such as CN-NPs [150], thiolated CN-NPs [151], NE [147,152], nanostructured lipid carriers [153], etc. These studies have verified that the appropriate formulation delivered by the intranasal route is beneficial to reduce the dose of Selegiline and enhance the brain bioavailability for the treatment of MDD.

Racemic tramadol hydrochloride is used clinically as a central analgesic. The (-) enantiomer of tramadol hydrochloride can inhibit the synaptic reuptake of monoamine neurotransmitters such as NE and 5-HT, increasing the concentration outside neurons. The thermoreversible gel loaded with tramadol hydrochloride CN-NPs prepared by Goyal et al. showed antidepressant potential by intranasal administration [154].

### 5.3. Peptides

Growing evidence suggests that impaired insulin signaling may contribute to both depression and type 2 diabetes. Intranasal delivery of antihyperglycemic agents can avoid systemic exposure-induced abnormal blood glucose levels, the BBB, and metabolically induced reductions in brain-targeted content [155]. The brain is an insulin-sensitive site. There are high densities of insulin receptors in the olfactory bulb, hypothalamus, hippocampus, and limbic system. Numerous studies have shown that insulin signaling may modulate hypothalamic–pituitary–adrenal axis homeostasis; affect levels of central neurotrophic factors and monoamine neurotransmitters; interact with gastrointestinal microbes; and improve mood, learning, and memory functions [156,157]. Oral insulin bioavailability is low. Insulin injected under the skin can lower healthy blood sugar levels and can even be life-threatening. The study found that intranasal delivery of insulin showed a 2000-fold increased AUC in the brain: plasma ratio compared with subcutaneous administration, with no apparent effect on blood glucose levels [158]. Lixisenatide acts as a GLP-1 receptor agonist to control blood sugar levels. It has neuroprotective effects in degenerative diseases such as Parkinson’s and Alzheimer’s. Wu et al. explored the possible mechanism of action of lixisenatide in the improvement of olfactory function and mood [159]. Intranasal lixisenatide not only improved depressive and anxious behaviors in a chronic unpredictable mild stress model, but also improved olfactory system function. In addition, intranasal lixisenatide was demonstrated to play an antidepressant role by regulating cyclic-AMP response binding protein (CREB)-mediated neurogenesis. In addition, intraventricular injection of glucagon 2 (GLP-2) showed a good antidepressant effect in depression model mice. Yamashita et al. combined a cell-penetrating peptide and a penetration-accelerating sequence with GLP-2 to prepare a GLP-2 derivative (PAS-CPP-GLP-2). PAS-CPP-GLP-2 can be smoothly taken up and released by nasal mucosa epithelial cells to enter the CNS. Studies have found that intranasal PAS-CPP-GLP-2 exhibited antidepressant effects similar to intracerebroventricular injection in mouse models but not intravenous injection [56,160]. In another studies, they developed a GLP-2-loaded nasal formulation prepared from polyoxyethylene (25) lauryl ether and β-cyclodextrin, which similarly increased the brain targeting of GLP-2 effects and antidepressant effects in a rat model [161].

Oxytocin is a peptide neurohormone composed of 9 amino acids synthesized by the hypothalamus and secreted by the posterior pituitary. Intranasal delivery of oxytocin has potential value in regulating anxiety and depression. Oxytocin is secreted by the pituitary into the peripheral circulation. It is involved in regulating the balance of the hypothalamic–pituitary–adrenal axis (HPA) and reducing corticosterone levels [162,163]. In addition, oxytocin is distributed in the hippocampus, amygdala, prefrontal cortex, cingulate cortex, olfactory bulb, and other brain regions involved in emotion regulation. Oxytocin can directly stimulate oxytocin receptors in the center nucleus and striatum to promote the release of 5-HT and dopamine [164,165,166]. Oxytocin also inhibits microglial activation, reduces the release of pro-inflammatory cytokines, and promotes neuronal plasticity. However, synthetic exogenous oxytocin has a short half-life and poor BBB permeability.

Brain-derived neurotrophic factor (BDNF) in the hippocampus and prefrontal cortex, which is closely related to depressive symptoms, was significantly reduced in patients with depression. BDNF plays an important role in the adaptive regulation of neuronal function (neuronal plasticity) [167,168]. Increased expression of BDNF in the brain may be an effective treatment for depression. Intracranial injection, microinjection, and peripheral delivery cause tissue damage and low bioavailability. Liu et al. fused TAT, which can improve permeability, and HA2, a hydrophobic peptide sequence that promotes lipid membrane stability, with BDNF, and loaded them onto mitochondrial virus (AAV) to successfully construct BDNF-HA2TAT/AAV through intranasal route. Western-blotting analysis showed that the content of BDNF in the hippocampus increased [169]. Compared with the control group and the AVV group, the BDNF-HA2TAT/AAV group significantly reversed the depressive behavior of the rats [170].

NAP is a peptide derived from activity-dependent neuroprotective protein, consisting of eight amino acids (Asn-Ala-Pro-V al-Ser-Ile-Pro-Gln, NAPVSIPQ). NAP functions by protecting the structural stabilization of neuronal microtubules, neuroprotection, and the role of axonal transport. Dang et al. constructed NT4-NAP/AAV to promote the expression of NAP in the CNS via the intranasal route. Intranasal administration largely solves the limitations of synthetic NAPs with short plasma half-life and weak ability to cross the BBB. Experiments have shown that the depressive symptoms of female mice are improved after ten days of administration [171,172].

Using the α2δ auxiliary subunit of V-gated Ca^2+^ channels and GABAA receptors as targets, Sukhanova et al. screened LCGA-17 in silico. LCGA-17 restores hippocampal NE levels and exhibits favorable anxiolytic and antidepressant properties in behavioral evaluations in animal models of rats. The cyclic neuropeptide Cortistatin-14 (CST-14) mRNA was significantly decreased after exposure to stressed mice. CST-14 exerts a rapid antidepressant effect through the regulation of ghrelin and GABA(A) systems [173]. Intranasal delivery of neuropeptide Y (NPY) demonstrated lower behavioral impairment compared with controls in a single chronic stress animal model [174]. Melanin-concentrating hormone (MCH) exerts anxiolytic and antidepressant effects by regulating the target of rapamycin (mTOR) signaling pathway. MCH can also restore proteins downregulated by stress on the synaptic surface [175]. 

Depression lowers blood and brain levels of neurotrophic factors (NTFs), which are restored by antidepressant treatment. Nerve growth factor (NGF) was first discovered and has regulatory effects on the development, differentiation, growth, regeneration, and functional properties of neurons. Intranasal administration of NGF reduces immobility time in the forced swim test and tail suspension test in mice. In addition, intranasal NGF increases glucose intake and activity time, and NE and dopamine release in the frontal cortex and hippocampus in rats after UCMS [176]. It also increases neurogenesis to compensate for cellular damage from depression.

The cytokine hypothesis reveals an inextricable link between depression and inflammatory responses. Studies have shown that the levels of pro-inflammatory cytokines such as interleukin-6 (IL-6) and tumor necrosis factor (TNF) in patients with depression are significantly increased [177]. Pro-inflammatory cytokines promote increased corticosterone synthesis, decreased monoamine neurotransmitter synthesis, and increased reuptake [178,179,180,181]. Shevela et al. reduced anxiety responses in mice in the field by intranasal delivery of soluble factors in M2 macrophage culture medium [182]. Concurrent studies have shown that M2 macrophages exert antidepressant potential by downregulating pro-inflammatory cytokines in the hippocampus, prefrontal cortex, and striatum.

### 5.4. Natural Active Ingredients

Albiflorin is a safe natural active component of monoterpene glycoside extracted from the root of Radix Paeoniae Alba. Albiflorin can act on multiple targets such as hippocampal phospholipids, tryptophan, and dopamine to play anti-inflammatory and antioxidative stress effects [183]. However, albiflorin is easily metabolized to benzoic acid by microorganisms in the gastrointestinal tract. After oral administration, the absolute intracerebral drug concentration is low and the absolute bioavailability is low [184,185]. Wang et al. prepared albiflorin-loaded alginate nanogels for nasal administration to avoid gastrointestinal degradation. Fluorescent labeling showed that albiflorin could quickly reach the brain for distribution after intranasal administration (≤30 min). The authors observed through tail suspension experiments in mice that low-dose intranasal administration significantly shortened the chronic unpredictable mild stress (CUMS) model of mice compared with intragastric gavage and intravenous injection of albiflorin solution. The reduction of pro-inflammatory cytokine levels and the repair of neuronal damage in CUMS rats further suggest that albiflorin has excellent potential for rapid antidepressant effects [186].

Berberine is a quaternary ammonium alkaloid biologically active ingredient extracted from Coptis chinensis with low toxicity and side effects. Berberine can reverse and improve the physiological changes caused by depression, such as monoamine neurotransmitter and dopamine levels, neuronal plasticity, and inflammatory responses [187,188]. Berberine has poor oral absorption, obvious intestinal first-pass metabolism, and limited absolute bioavailability [189]. Cui et al. improved the solubility of berberine by inclusion of hydroxypropyl-β-cyclodextrin (HP-β-CD). The encapsulated drug was then loaded into a thermosensitive hydrogel for intranasal administration. The relative intracerebral bioavailability of berberine showed that the intranasal formulation of berberine was 110 times higher than the oral inclusion complex of berberine–cyclodextrin. Pharmacological studies have found that the intranasal route, in addition to increasing the levels of monoamine neurotransmitters in the hippocampus compared with oral administration, exhibits a potential antidepressant mechanism by restoring sphingolipid and phospholipid abnormalities and mitochondrial dysfunction [190]. In another study, they combined two natural bioactive ingredients with antidepressant effects, berberine and evodiamine, into a nasal formulation. The bioavailability of intranasal hydrogels was more than 100 times higher than that of gavage drug solutions. Further, the intranasal formulation significantly improved behavioral despair by modulating monoamine levels and related metabolic pathways in mice [191].

Cang-ai volatile oil is a Chinese herbal volatile oil that has been used clinically as an intranasal inhaler. Studies have shown that Cang-ai volatile oil can inhibit microglia activation and kynurenine pathway to regulate 5-HT and play an antidepressant effect [192]. The forced swim test, open field test, sucrose preference test, etc. confirmed that intranasal delivery of Cang-ai volatile oil can effectively regulate the metabolism of dopamine and 5-HT in the brain of CUMS rats and improve depressive behavior [193].

Icariin (ICA), the main component of Epimedium grandiflorum, has shown therapeutic effects in osteoporosis, tumor, inflammation, cardiovascular disease, and even depression [194]. However, pharmacokinetic studies of ICA have shown poor oral absorption and brain bioavailability [195]. Wang Q. S. and Cui Y. L. et al. prepared an ICA nanogel loaded self-assembled thermosensitive hydrogel system (ICA-NGSTH) to treat depression by intranasal administration. ICA-NGSTH changes from a solid phase to a liquid phase in the nasal cavity and adheres to the nasal mucosa, thereby prolonging the residence time and releasing the drug slowly and continuously. They used fluorescence imaging of rhodamine B-labeled nanogels to study the in vivo distribution. ICA-NGSTH could be distributed in the brain in about half an hour and showed zero order kinetic release within 10 h. By comparing the oral route of ICA, intranasal ICA-NGSTH showed better behavior in an animal model of depression [196].

Olfactory dysfunction is a complication of a variety of CNS disorders, including depression. Gao et al. investigated the effects of white tea extracts on depressive behavior and olfactory disturbances in CUMS mice by intranasal administration. High and low levels of white tea extracts could effectively reverse depressive behavior in mice. Olfactory avoidance tests and olfactory sensitivity tests showed their relief of olfactory dysfunction. Pharmacological studies found that white tea reduced mitochondrial and synaptic damage in the olfactory bulb and enhanced the content of BDNF (Table 1) [197].

## 6. Summary and Outlook

Under the action of different factors, the incidence of depression in today’s society is becoming more and more serious. Drug treatment for depression is mainly oral administration, which is challenged by extensive first-pass metabolism, the BBB, and systemic side effects. Some antidepressant active ingredients, such as peptides, natural active ingredients, etc., cannot achieve their high brain bioavailability due to the limitations of their own physical and chemical properties. The direct pathway between the nasal cavity and the brain provides a reliable guarantee for improving the bioavailability of antidepressant active ingredients and reducing side effects. As the olfactory system highly overlaps with areas that process emotion and memory functions, intranasal administration (especially to the olfactory area) may be a potential route for treating depression. Antidepressant active ingredients can enter the brain directly through the olfactory sensory nerve, trigeminal nerve, and olfactory mucosal epithelial pathways, or indirectly through the rich capillaries in the respiratory area and lymphatic tissue.

With the continuous progress of medical technology and the pharmaceutical engineering industry, remarkable research achievements have been made in how to make more effective use of nose-to-brain drug delivery, but there are still some problems to be solved. The small size of the nasal space and olfactory area limits the amount of drug that needs to be delivered directly to the brain. Moreover, factors such as the close connection of olfactory epithelial cells, the clearance of nasal mucosa cilia and the degradation of antidepressant active ingredients by various enzymes all make fewer parts to be absorbed; so, the method of enhancing absorption is very important. At present, some remarkable achievements have been made in the research of promoting nasal mucous absorption. For example, some materials with good biocompatibility, biodegradation, and low toxicity, such as permeability enhancers, adhesives, enzyme inhibitors, and nanoparticles, have been well applied in promoting nose-to-brain delivery. Solvent enhancers, antioxidants, preservatives, moisturizers, buffers, and taste maskers are added to the formulation to ensure drug stability and patient compliance. However, the treatment of depression is often long-term, and these absorption-based materials can more or less cause irritation and side effects to the nasal cavity and other tissues and organs. On the other hand, the invention of some nasal delivery devices, such as Vianase™, has significantly improved the deposition of antidepressant active ingredients in the olfactory region of the upper nasal cavity compared with traditional delivery devices. However, the deposition rate in the olfactory area is still relatively low; the highest is only about 50%. Therefore, there is an urgent need to find better excipients and new devices for enhancing drug delivery in the nose and brain.

Current studies on intranasal administration of antidepressant active ingredients are mostly limited to the cellular level and model animal level. In addition, different stimulation methods may also lead to individual differences in the model. There are differences between the structure and physiological conditions of the human nasal cavity and experimental animals; thus, it is not effective in an animal model but in the human body. To further verify the drug, a clinical observation test is needed to evaluate its safety and effectiveness. In addition to intranasal administration, the development of new routes of administration, such as transdermal targeted administration, to overcome the disadvantages of oral and injectable administration is also one of the main exploration directions to improve the efficacy of antidepressant treatment in the future.

## Figures and Tables

**Table 1:** Summary of studies on antidepressant active ingredients and new dosage forms for intranasal administration.

Types	Ingredients	Dosage Form	Characterization	Ex Vivo/In Vivo Studies	Relevant Outcomes	Ref.
Antidepressants	Venlafaxine	Poly lactic-co-glycolic acid nanoparticles (PLGA-NPs); Peptide-modified nanoparticles	PS = 206.3 ± 3.7 nm; PI = 0.041 ± 0.017; ZP = −26.5 ± 0.5 mV; around 200 nm after lyophilization process; DL = 10–12%; EE = 48–50%.	In vitro cell viability and cellular uptake (hCMEC/D3 cells); Permeability assay and transport studies; Biodistribution studies (C57/bl6 mice).	Cell viability of h-CMEC/D3 cells is more than 85% in the MTT assay. In vivo biodistribution studies showed higher concentrations of plain fluorescent NPs than functionalized NPs in the brain after 30 min of administration.	[107]
		Chitosan nanoparticles (CN-NPs)	PS = 167 ± 6.5 nm; PI = 0.367 ± 0.045; ZP = +23.83 ± 1.76 mV; DL = 32.25 ± 1.63%; EE = 79.3 ± 2.6%; Yield = 71.42 ± 3.24%.	Ex vivo permeation studies using porcine nasal mucosal membrane (Franz cells); Pharmacodynamic studies in Wistar rats (modified forced swim test, locomotor activity test); Qualitative localization and biodistribution studies by confocal laser scanning microscopy; Pharmacokinetic analysis.	The cumulative drug permeability after 24 h in VLF CN-NPs was nearly 3 times compared with VLF solution. VLF CN-NPs showed a more significant antidepressant effect than VLF solution on chronic depression rats by forced swimming method. DTE (%)/DTP (%): NPs = 508.59/80.34	[108]
	Desvenlafaxine	Chitosan-coated poly lactic-co-glycolic acid nanoparticles (PLGA-CN-NPs)	PS = 172.5 ± 10.2 nm; PI = 0.254 ± 0.02; ZP = +35.63 ± 8.25 mV; DL = 30.8 ± 3.1%; EE = 76.4 ± 4.2%; Release (24 h) = 77.21 ± 3.87% (pH 7.4) and 76.32 ± 3.54% (pH 6.0).	Ex vivo permeation studies on porcine nasal mucosa; Pharmacodynamic studies (Wistar rats); Stress-induced model (forced swimming test); Drug-induced model (reserpine reversal test); Biochemical estimation of serotonin, noradrenaline, and dopamine; Blood and brain pharmacokinetic studies.	In a rodent model of depression, compared with intranasal DVF solution and oral administration, increased levels of 5-HT and NE in the brain showed a more pronounced antidepressant effect. Pharmacokinetic parameters such as concentration, half-life, and AUC in the brain after intranasal administration were higher than those of through intravenous. DTE (%)/DTP (%) = 544.23/81.62 (DVF-NP s) DTE (%)/DTP (%) = 202.41/50.59 (DVF)	[111]
	Agomelatine	Nanoemulsion thermosensitive in situ gel + 0.5% Chitosan	Gelling point = 28 ± 1 °C; Mucoadhesive strength = 6246.27 dynes/cm^2^; NEs: PS = 206.3 ± 3.7 nm; Micelles of P-407: PS = 142.58 ± 4.21 nm; Ago-NE-gel + 0.5%chitosan: Viscosity = 2439 ± 23 cP (35 ± 1 °C); pH = 5.8 ± 0.2.	In vitro gel erosion study; Ex vivo drug permeation through the bovine nasal mucosa; Nasal toxicity study; Pharmacokinetic analysis: DTE (%) and DTP (%); Pharmacodynamic studies (Behavioral test; modified forced swim test and tail suspension test).	Pharmacokinetic study in Wistar rats showed plasma concentration in the brain was 2.82 times higher than that of the intravenous suspension via the intranasal route. DTE (%)/DTP (%) = 344.9/71.0	[112]
		Solid lipid nanoparticles (SLNs)	PS = 167.70 ± 0.42 nm; PI = 0.12 ± 0.10; ZP = −17.90 ± 2.70 mV; EE = 91.25 ± 1.70%; Release (1 h)/(8 h) = 35.40 ± 1.13%/80.87 ± 5.16%.	Pharmacokinetic study (rats): assay of agM in plasma and brain; Pharmacokinetic analysis: DTE (%) and DTP (%).	The nasal solid lipid nanoparticles prepared by Ahmed et al. were superior to the oral suspension in brain concentration, AUC_0–360min_, and absolute bioavailability (44.44%) DTE (%)/DTP (%) = 190.02/47.37	[113]
	Duloxetine	Nanostructured lipid carriers (NLCs)	PS = 137.2 ± 2.88 nm; ZP = -31.53 ± 11.21 mV; DL = 9.73 ± 3.22%; EE = 79.15 ± 4.17%.	Biodistribution studies (Wistar rats); Pharmacokinetic study; Gamma-imaging study.	Intranasal DLX-NLCs showed higher concentrations in blood and brain compared with DLX solution and oral route, which showed the same results in behavioral tests in mice. Intranasal NLCs were 8-fold higher in brain concentrations than intravenous DLX. DTE (%)/DTP (%) = 757.74/86.80 (DLX-NLC) DTE (%)/DTP (%) = 287.34/65.12 (DLX)	[116,117]
		Thiomer gel loaded with proniosomes	20% *w/v* PF127, 5% *w/v* PF68; 3.76 lipid ratio; PS = 265.13 ± 9.85 nm; GT = 32 ± 0.05 °C; EE = 98.13 ± 0.50%; Release (3 h) = 33%.	Pharmacokinetic analysis: DTE (%) and DTP (%); Stability study.	Thiomer gel loaded with duloxetine proniosomes increased the retention time and sustained release and penetration of DLX in the nasal mucosa (1.96 times that of duloxetine proniosomes). DTE (%)/DTP (%) = 137.77/10.5	[118]
	Paroxetine	Nanoemulsion (NEs)	PS = 58.47 ± 3.02 nm; PDI = 0.339 ± 0.007; ZP = −33 mV; Transmittance = 100.60 ± 0.577%; Refractive index = 1.412 ± 0.003.	Ex vivo permeation studies using porcine nasal mucosal membrane (Franz cells); Pharmacodynamic studies (Wistar rats; forced swimming test, locomotor activity test); Biochemical estimation: GSH and TBARS.	The permeability of paroxetine NEs was 2.57 times higher than that of its suspension via permeation studies. Results of behavioral studies in rats showed that intranasal administration of paroxetine NEs significantly improved behavioral activity in depressed rats compared with the oral suspension of paroxetine.	[121]
	Trazodone	Microemulsion	labelling yield = 91.23 ± 2.12%; In vitro stability of ^131^I-TZ = 6 h; Droplet size = 16.4 ± 2.5 nm; PDI = 0.11 ± 0.02; ZP = 3.83 ± 0.36; Viscosity (25 °C) = 261.7 ± 3.0; Viscosity (37 °C) = 157.3 ± 7.5.	Biodistribution of ^131^I-TZ; The ^131^I-TZ uptake in organs and body fluids.	Sayyed et al. radiolabeled trazodone and compared the pharmacokinetic parameters of intranasal delivery of ^131^I-TZ solution, ^131^I-TZ microemulsion, and intravenous injection of ^131^I-TZ solution. Intranasal ^131^I-TZ microemulsion had sustained and higher brain uptake at any time tested than the other two formulations and routes. In addition, the blood exposure of intranasal ^131^I-TZ microemulsion was lower than that of intravenous injection, reducing systemic toxicity.	[123]
	Quetiapine fumarate	Microemulsion Chitosan microemulsion (CH-ME) methyl-β-cyclodextrin microemulsion (MeβCD-ME)	PS: QF-ME = 29.75 ± 0.99 nm; CH-ME = 35.31 ± 1.71 nm; MeβCD-ME = 46.55 ± 1.9 nm with; PDI: QF-ME = 0.221 ± 0.01; CH-ME = 0.249 ± 0.03; MeβCD-ME = 0.233 ± 0.02; ZP: QF-ME = 2.77 ± 0.51; CH-ME = 20.29 ± 1.23 MeβCD-ME = 8.43 ± 0.7; Viscosity: QF-ME = 17.5 ± 0.69 cP; CH-ME = 38.5 ± 1.26 cP; MeβCD-ME = 33.3 ± 0.93 cP.	Ex vivo mucoadhesive strength; Ex vivo nasal and intestinal diffusion study (goat nasal mucosa and small intestine); Nasal mucosal toxicity test; Pharmacokinetic analysis: DTE (%) and DTP (%).	The brain bioavailability of quetiapine fumarate of chitosan-coated microemulsion was 3.8-fold and 2.7-fold higher than that of drug solution and chitosan-free microemulsion, respectively. DTE (%)/DTP (%) = 371.20 ± 12.02/ 68.66 ± 6.84 (QF-ME) DTE (%)/DTP (%) = 453.69 ± 10.17/80.51 ± 6.46 (CH-ME)	[125]
	Doxepin hydrochloride	Thermoreversible biogels	Gelation temperature = 37.4 °C; Gelation time = 7.32 min pH = 6.93.	In vitro penetration test on sheep nasal mucosa; Stress-induced model (forced swimming test).	Compared with doxepin hydrochloride solution, the thermoreversible biogel showed more advantages in immobility time and swimming activity count in mice after 13 days of drug administration.	[126]
Off-label drugs	Ketamine/Esketamine	Nasal spray	N/A	N/A	Ketamine, whether administered intravenously or intranasally, has a higher bioavailability than the oral route, and has a more rapid and significant effect than traditional antidepressants with delayed onset of action. Due to the plasma elimination half-life of ketamine of 2–4 h and the discomfort associated with invasive administration, delivery of ketamine directly to the brain via the nasal cavity is a more advantageous strategy.	[127,128]
	Amisulpride	Lipid-based poloxamer-gellan gum nanoemulgel AMS nanoemulsion (AMS-NE) AMS in situ nanoemulgel (AMS-NG)	AMS-NE: PS = 92.15 ± 0.42 nm; PI = 0.46 ± 0.03; ZP = −18.22 mV; Transmittance = 99.57%; Mucoadhesive strength = 1.24 g; Release (4 h) = 99.99%; AMS-NG: PS = 106.11 ± 0.26 nm; PI = 0.51 ± 0.01; ZP = −16.01 mV; Transmittance = 98.47%; Mucoadhesive strength = 8.90 g; Release (4 h) = 98.96%.	Ex vivo drug permeation study on freshly isolated sheep nasal mucosa; In vivo animal experiments (pharmacokinetic study, AMS in brain and blood plasma samples); Animal behavioral studies (induced locomotor activity test, paw test); In vivo safety assessment.	Pharmacokinetic studies in Wister rats showed that the intranasal C(max) of the brain was 3.39 times higher than that of the intravenous administration and intranasal administration within one month did not affect blood leukocyte and granulocyte counts. DTE (%)/DTP (%) = 314.08/76.13 (AMS-NE) DTE (%)/DTP (%) = 1821.72/275.09 (AMS-NG)	[139]
	Aripiprazole	Mucoadhesive nanoemulsion	PS = 121.8 ± 1.5 nm; PI = 0.248 ± 0.05; ZP = −18.89 ± 3.47 mV; Viscosity = 187.79 ± 5.35 cP (25% Carbopol); Viscosity = 626.32 ± 8.63 cP (1% Carbopol); Release (8 h) = 84.92%.	Ex vivo permeation test and nasal ciliotoxicity on sheep nasal mucosa; In vitro cytotoxicity study (Vero cells, PC12 cells); In vivo pharmacokinetic study (DTE (%) and DTP (%)); Locomotor activity study.	Pharmacokinetic studies with single-dose administration showed that the plasma concentration in the brain of intranasal ARP-MNE was 1.44 and 6.03 times higher than that of intranasal and intravenous ARP-NE, respectively, and the Tmax was smaller than that of intravenously administered ARP-NE. DTE (%)/ DTP (%) = 96.90/89.73	[144]
		Poly(caprolactone) nanoparticles	PS = 199.2 ± 5.65 nm; ZP = -21.4 ± 4.6 mV; EE = 69.2 ± 2.34%; Release (8 h) = 90 ± 2.69%.	Ex vivo diffusion studies on goat nasal mucosa; Nasal toxicity study (goat nasal mucosa); In vivo pharmacokinetics study (DTE (%) and DTP (%)).	The AUC_0–8h_ of Aripiprazole in the rat brain administered by the intranasal route of APNPs was approximately twice that of the intravenous route. DTE (%)/DTP (%) = 64.11/74.34	[145]
	Selegiline	Chitosan nanoparticle	PS = 341.6 ± 56.91 nm; PI = 0.317 ± 0.29; ZP = −13.4 ± 0.04 mV; EE = 92.20 ± 7.15%; Release (8 h) = 90 ± 2.69%.	Ex vivo drug diffusion on sheep nasal mucosa; Pharmacokinetics and pharmacodynamics studies; Behavioral testing; Biochemical analyses: dopamine level, catalase activity, reduced glutathione (GSH) content.	The Cmax of plain solution of selegiline in the brain and plasma by intranasal administration (Tmax = 5 min) was 20 and 12 times higher, respectively, compared with oral administration (Tmax = 15 min). Furthermore, intranasal administration of selegiline-loaded CN-NPs and mucoadhesive thermosensitive gel showed superior formulation advantages compared with the AUC_0–24h_ of plain solution.	[148,149]
Peptides	Insulin	N/A	N/A	Pharmacokinetics study (insulin concentrations in brain and plasma via different delivery routes); AUC_brain: plasma_ ratio; Repeated in insulin administration.	The study found intranasal delivery of insulin showed a 2000-fold increased AUC_brain: plasma_ ratio compared with subcutaneous administration, with no apparent effect on blood glucose levels.	[158]
	Lixisenatide	N/A	N/A	Chronic unpredictable mild stress depression model (rats); Behavioral studies (forced swim test, tail suspension test, open field test); Cells were labeled with BrdU and neurogenesis in the olfactory bulb and hippocampus was observed.	Intranasal lixisenatide not only improved depressive and anxious behaviors in a chronic unpredictable mild stress model, but also improved olfactory system function. In addition, intranasal lixisenatide was demonstrated to play an antidepressant role by regulating cyclic-AMP response binding protein (CREB)-mediated neurogenesis.	[159]
	GLP-2	PAS-CPPs-GLP-2	N/A	Behavioral studies (forced swim test, tail suspension test, open field test); Distribution test (rats’ brain).	Studies have found that intranasal PAS-CPP-GLP-2 exhibited antidepressant effects similar to intracerebroventricular injection in mouse models, but not intravenous injection.	[56,160]
	BDNF	BDNF-HA2TAT/AAV	Each step was qualified by specific restriction enzyme reactions and AGE; High expression of BDNF in infected Hela cells.	Chronic unpredictable mild stress depression model (rats); Behavioral assessment (forced swim test, sucrose preference test, open field test); Body weight; Western-blotting analysis; Expression of BDNF mRNA.	Western-blotting analysis showed that the content of BDNF in the hippocampus increased via intranasal administration. Compared with the control group and the AVV group, the BDNF-HA2TAT/AAV group significantly reversed the depressive behavior of the rats.	[169,170]
	NAP	NT4-NAP/AAV	Each step was qualified by specific restriction enzyme reactions and AGE; Expression of BDNF in infected PC12 cells.	Behavioral assessment (forced swim test, sucrose preference test, open field test); Effect on plasma CORT; Expression of 5-HT and BNDF in hippocampus.	Experiments have shown that the depressive symptoms of female mice are improved after ten days of administration. Although the effect is not significant, it also proves that intranasal administration from different targets, such as microtubules, provide new ideas for the treatment of depression.	[171,172]
	NPY/LCG-17/MCH/CST-14/NGF	N/A	N/A	Behavioral assessment (forced swim test, sucrose preference test, open field test); Biochemical studies.	These peptides bypass the blood–brain barrier via a non-invasive intranasal route of administration, improving bioavailability and brain targeting. The peptides both improve anxiety and depression behavior in animal models. The peptides also promote neuroplasticity in the central nervous system, especially the hippocampus and prefrontal cortex.	[173,174,175,176]
Natural active ingredients	Albiflorin	Alginate nanogels	PS = 45.6 ± 5.2 nm; PI < 0.20; ZP = −19.8 ± 0.9 mV; EE = ±7.15%; Release (12 h) = 99%; Gelling temperature = 28 °C.	In vivo fluorescence distribution analysis of alginate nanogels (rats); Pharmacodynamic study; Antidepressant behavioral studies: tail suspension test; Transcriptome studies: cAMP, calcium ion, and cGMP PKG signal pathway.	Fluorescent labeling showed that albiflorin could quickly reach the brain for distribution after intranasal administration (≤30 min). The authors observed through tail suspension experiments in mice that low-dose intranasal administration significantly shortened the chronic unpredictable mild stress model of mice compared with intragastric gavage and intravenous injection of albiflorin solution. Do not move time. The reduction of pro-inflammatory cytokine levels and the repair of neuronal damage in CUMS rats further suggest that albiflorin has an excellent potential for rapid antidepressant effects.	[186]
	Berberine	Cyclodextrin + thermosensitive hydrogel	The berberine /HP-β-CD inclusion complex (^1^H-NMR-NMR showed good degree of inclusion); Gelling temperature = 30 °C; Release (6 h) = 83.29 ± 3.98%; Loading efficiency = 22.86%.	Brain targeting of berberine study (Radioactive tracer of ^125^I); Pharmacokinetic analysis: berberine in hippocampus; Monoamine neurotransmitters in rats (reserpine-induced model).	The relative intracerebral bioavailability of berberine showed that the intranasal formulation of berberine was 110 times higher than the oral inclusion complex of berberine–cyclodextrin. Pharmacological studies have found that the intranasal route, in addition to increasing the levels of monoamine neurotransmitters in the hippocampus compared with oral administration, exhibits a potential antidepressant mechanism by restoring sphingolipid and phospholipid abnormalities and mitochondrial dysfunction.	[190]
	Berberine and Evodiamine	Thermosensitive in situ hydrogels	P407/P188/HP-β-CD/PEG 8000 = 20/0/8/1; Release = 93% (berberine); Release = 43% (evodiamine); Gelling temperature = 28 °C.	Pharmacokinetic study (plasma and hippocampus); Antidepressant behavioral studies (open field test, tail suspension test); Monoamine neurotransmitters studies in rats.	The bioavailability of intranasal hydrogels was more than 135- and 112-fold higher than that of gavage berberine and evodiamine solutions. The intranasal formulation significantly improved behavioral despair by modulating monoamine levels and related metabolic pathways in mice.	[191]
	Cang-ai volatile oil	Intranasal inhaler	N/A	Chronic unpredictable mild stress depression model (rats); Behavioral studies (open field test, forced swim test, and sucrose preference test); Expression of pro-inflammatory cytokines and monoamine neurotransmitters studies in prefrontal cortex.	Studies have shown that Cang-ai volatile oil can inhibit microglia activation and kynurenine pathway to regulate 5-HT and play an antidepressant effect. The forced swim test, open field test, sucrose preference test, etc. confirmed that intranasal delivery of Cang-ai volatile oil can effectively regulate the metabolism of dopamine and 5-HT in the brain of CUMS rats and improve depressive behavior.	[192,193]
	Icariin	Nanogel loaded thermosensitive hydrogel (NGSTH)	PS = 73.80 ± 2.34 nm; PI < 0.15; ZP = −19.2 ± 1.14 mV; Loading efficiency = 2.03%; Release (36 h) = 70% (nanogel); Gelling temperature = 30 °C; Release (36 h) = 100% (NGSTH).	In vivo distribution fluorescently labeled nanogels Behavioral testing (tail suspension test, forced swim test); Expression of pro-inflammatory cytokines and morphological changes in the hippocampus.	ICA-NGSTH could be distributed in the brain in about half an hour and showed zero order kinetic release within 10 h. By comparing the oral route of ICA, intranasal ICA-NGSTH showed better behavior improvement ability in an animal model of depression.	[196]
	White tea	N/A	N/A	Chronic unpredictable mild stress depression model (rats); Behavioral testing (open-field test, sucrose preference test, buried food pellet test); Olfactory sensitivity test.	High and low levels of white tea extracts could effectively reverse depressive behavior in mice. Olfactory avoidance tests and olfactory sensitivity tests showed its relief of olfactory dysfunction. Pharmacological studies found that white tea reduced mitochondrial and synaptic damage in the olfactory bulb and enhanced the content of BDNF.	[197]

Abbreviations: PS, globule size; ZP, zeta potential; PI, polydispersity index; DTE (%), drug targeting efficiency; DTP (%), nose-to-brain direct transport percentage; BDNF, brain-derived neurotrophic factor; CORT, corticosterone; P407, poloxamer 407; P188, poloxamer 188.

## References

[1] Evans-Lacko S., Aguilar-Gaxiola S., Al-Hamzawi A., Alonso J., Benjet C., Bruffaerts R., Chiu W.T., Florescu S., de Girolamo G., Gureje O. (2017). Socio-economic variations in the mental health treatment gap for people with anxiety, mood, and substance use disorders: Results from the WHO World Mental Health (WMH) surveys. Psychol. Med..

[2] Panek M., Kawalec P., Pilc A., Lasoń W. (2020). Developments in the discovery and design of intranasal antidepressants. Expert Opin. Drug Discov..

[3] Depression and Other Common Mental Disorders: Global Health Estimates. https://www.who.int/publications/i/item/depression-global-health-estimates.

[4] Illum L. (2000). Transport of drugs from the nasal cavity to the central nervous system. Eur. J. Pharm. Sci..

[5] Kashyap K., Shukla R. (2019). Drug Delivery and Targeting to the Brain Through Nasal Route: Mechanisms, Applications and Challenges. Curr. Drug Deliv..

[6] Mato Y.L. (2019). Nasal route for vaccine and drug delivery: Features and current opportunities. Int. J. Pharm..

[7] Giunchedi P., Gavini E., Bonferoni M.C. (2020). Nose-to-brain delivery. Pharmaceutics.

[8] O’Leary O.F., Dinan T.G., Cryan J.F. (2015). Faster, better, stronger: Towards new antidepressant therapeutic strategies. Eur. J. Pharmacol..

[9] Pardridge W.M. (2016). CSF, blood-brain barrier, and brain drug delivery. Expert Opin. Drug Deliv..

[10] Daneman R., Prat A. (2015). The blood-brain barrier. Cold Spring Harb. Perspect. Biol..

[11] Abbott N.J., Patabendige A.A.K., Dolman D.E.M., Yusof S.R., Begley D.J. (2010). Structure and function of the blood-brain barrier. Neurobiol. Dis..

[12] Zheng Y., Chen X., Benet L.Z. (2015). Reliability of In Vitro and In Vivo Methods for Predicting the Effect of P-Glycoprotein on the Delivery of Antidepressants to the Brain. Clin. Pharmacokinet..

[13] O’Brien F.E., Dinan T.G., Griffin B.T., Cryan J.F. (2011). Interactions between antidepressants and P-glycoprotein at the blood-brain barrier: Clinical significance of in vitro and in vivo findings. Br. J. Pharmacol..

[14] Brückl T.M., Uhr M. (2016). *ABCB1* genotyping in the treatment of depression. Pharmacogenomics.

[15] Bicker J., Fortuna A., Alves G., Falcão A. (2020). Nose-to-brain delivery of natural compounds for the treatment of central nervous system disorders. Curr. Pharm. Des..

[16] Long Y., Yang Q., Xiang Y., Zhang Y., Wan J., Liu S., Li N., Peng W. (2020). Nose to brain drug delivery—A promising strategy for active components from herbal medicine for treating cerebral ischemia reperfusion. Pharmacol. Res..

[17] Shringarpure M., Gharat S., Momin M., Omri A. (2020). Management of epileptic disorders using nanotechnology-based strategies for nose-to-brain drug delivery. Expert Opin. Drug Deliv..

[18] Wang Z., Xiong G., Tsang W.C., Schätzlein A.G., Uchegbu I.F. (2019). Nose-to-brain delivery. J. Pharmacol. Exp. Ther..

[19] Kim B.-Y., Bae J.H. (2022). Olfactory Function and Depression: A Meta-Analysis. Ear Nose Throat J..

[20] Staszelis A., Mofleh R., Kocsis B. (2022). The effect of ketamine on delta-range coupling between prefrontal cortex and hippocampus supported by respiratory rhythmic input from the olfactory bulb. Brain Res..

[21] Schwartz J.S., Tajudeen B.A., Kennedy D.W. (2019). Diseases of the nasal cavity. Handb. Clin. Neurol..

[22] Gonçalves J., Alves G., Fonseca C., Carona A., Bicker J., Falcão A., Fortuna A. (2021). Is intranasal administration an opportunity for direct brain delivery of lacosamide?. Eur. J. Pharm. Sci..

[23] Dhuria S.V., Hanson L.R., Frey W.H. (2010). Intranasal delivery to the central nervous system: Mechanisms and experimental considerations. J. Pharm. Sci..

[24] Samaridou E., Alonso M.J. (2018). Nose-to-brain peptide delivery—The potential of nanotechnology. Bioorg. Med. Chem..

[25] Rottstädt F., Han P., Weidner K., Schellong J., Wolff-Stephan S., Strauß T., Kitzler H., Hummel T., Croy I. (2018). Reduced olfactory bulb volume in depression-A structural moderator analysis. Hum. Brain Mapp..

[26] Rottstaedt F., Weidner K., Strauß T., Schellong J., Kitzler H., Wolff-Stephan S., Hummel T., Croy I. (2017). Size matters—The olfactory bulb as a marker for depression. J. Affect. Disord..

[27] Cecon E., Ivanova A., Luka M., Gbahou F., Friederich A., Guillaume J., Keller P., Knoch K., Ahmad R., Delagrange P. (2018). Detection of recombinant and endogenous mouse melatonin receptors by monoclonal antibodies targeting the C-terminal domain. J. Pineal Res..

[28] Noseda A.C.D., Rodrigues L.S., Targa A.D.S., Ilkiw J.L., Fagotti J., Dos Santos P.D., Cecon E., Markus R.P., Solimena M., Jockers R. (2021). MT(2) melatonin receptors expressed in the olfactory bulb modulate depressive-like behavior and olfaction in the 6-OHDA model of Parkinson's disease. Eur. J. Pharmacol..

[29] Crowe T.P., Greenlee M.H.W., Kanthasamy A.G., Hsu W.H. (2018). Mechanism of intranasal drug delivery directly to the brain. Life Sci..

[30] Renner D.B., Svitak A.L., Gallus N.J., Ericson M.E., Frey W.H., Hanson L.R. (2012). Intranasal delivery of insulin via the olfactory nerve pathway. J. Pharm. Pharmacol..

[31] Tan M.S.A., Parekh H.S., Pandey P., Siskind D.J., Falconer J.R. (2020). Nose-to-brain delivery of antipsychotics using nanotechnology: A review. Expert Opin. Drug Deliv..

[32] Altner H., Altner-Kolnberger I. (1974). Freeze-fracture and tracer experiments on the permeability of the zonulae occludentes in the olfactory mucosa of vertebrates. Cell Tissue Res..

[33] Durante M.A., Kurtenbach S., Sargi Z.B., Harbour J.W., Choi R., Kurtenbach S., Goss G.M., Matsunami H., Goldstein B.J. (2020). Single-cell analysis of olfactory neurogenesis and differentiation in adult humans. Nat. Neurosci..

[34] Trevino J., Quispe R., Khan F., Novak V. (2020). Non-Invasive Strategies for Nose-to-Brain Drug Delivery. J. Clin. Trials.

[35] Lochhead J.J., Thorne R.G. (2012). Intranasal delivery of biologics to the central nervous system. Adv. Drug Deliv. Rev..

[36] Croy I., Hummel T. (2020). Involvement of nasal trigeminal function in human stereo smelling. Proc. Natl. Acad. Sci. USA.

[37] Lochhead J.J., Kellohen K.L., Ronaldson P.T., Davis T.P. (2019). Distribution of insulin in trigeminal nerve and brain after intranasal administration. Sci. Rep..

[38] Kumar N.N., Lochhead J., Pizzo M., Nehra G., Boroumand S., Greene G., Thorne R.G. (2018). Delivery of immunoglobulin G antibodies to the rat nervous system following intranasal administration: Distribution, dose-response, and mechanisms of delivery. J. Control. Release.

[39] Pang Y., Fan L.-W., Carter K., Bhatt A. (2019). Rapid transport of insulin to the brain following intranasal administration in rats. Neural Regen. Res..

[40] Lochhead J., Wolak D.J., Pizzo M., Thorne R.G. (2015). Rapid Transport within Cerebral Perivascular Spaces Underlies Widespread Tracer Distribution in the Brain after Intranasal Administration. J. Cereb. Blood Flow Metab..

[41] Iliff J.J., Wang M., Liao Y., Plogg B.A., Peng W., Gundersen G.A., Benveniste H., Vates G.E., Deane R., Goldman S.A. (2012). A Paravascular Pathway Facilitates CSF Flow Through the Brain Parenchyma and the Clearance of Interstitial Solutes, Including Amyloid β. Sci. Transl. Med..

[42] Pardeshi C.V., Rajput P.V., Belgamwar V.S., Tekade A.R. (2011). Formulation, optimization and evaluation of spray-dried mucoadhesive microspheres as intranasal carriers for Valsartan. J. Microencapsul..

[43] Gänger S., Schindowski K. (2018). Tailoring Formulations for Intranasal nose-to-brain delivery: A review on architecture, physico-chemical characteristics and mucociliary cearance of the nasal olfactory mucosa. Pharmaceutics.

[44] Schwarz B., Merkel O.M. (2019). Nose-to-brain delivery of biologics. Ther. Deliv..

[45] Smith T.D., Bhatnagar K.P. (2019). Anatomy of the olfactory system. Handb. Clin. Neurol..

[46] Olivares J., Schmachtenberg O. (2019). An update on anatomy and function of the teleost olfactory system. PeerJ.

[47] Palleria C., Roberti R., Iannone L.F., Tallarico M., Barbieri M.A., Vero A., Manti A., De Sarro G., Spina E., Russo E. (2019). Clinically relevant drug interactions between statins and antidepressants. J. Clin. Pharm. Ther..

[48] Wyska E. (2019). Pharmacokinetic considerations for current state-of-the-art antidepressants. Expert Opin. Drug Metab. Toxicol..

[49] Erdő F., Bors L.A., Farkas D., Bajza Á., Gizurarson S. (2018). Evaluation of intranasal delivery route of drug administration for brain targeting. Brain Res. Bull..

[50] Ruigrok M.J., de Lange E.C. (2015). Emerging insights for translational pharmacokinetic and pharmacokinetic-pharmacodynamic studies: Towards prediction of nose-to-brain transport in humans. AAPS J..

[51] Martins P.P., Smyth H.D., Cui Z. (2019). Strategies to facilitate or block nose-to-brain drug delivery. Int. J. Pharm..

[52] Iwasaki S., Yamamoto S., Sano N., Tohyama K., Kosugi Y., Furuta A., Hamada T., Igari T., Fujioka Y., Hirabayashi H. (2019). Direct Drug Delivery of Low-Permeable Compounds to the Central Nervous System Via Intranasal Administration in Rats and Monkeys. Pharm. Res..

[53] Marttin E., Verhoef J.C., Merkus F.W.H.M. (1998). Efficacy, Safety and Mechanism of Cyclodextrins as Absorption Enhancers in Nasal Delivery of Peptide and Protein Drugs. J. Drug Target..

[54] Li Y., Li J., Zhang X., Ding J., Mao S. (2014). Non-ionic surfactants as novel intranasal absorption enhancers: In vitro and in vivo characterization. Drug Deliv..

[55] Rassu G., Soddu E., Cossu M., Brundu A., Cerri G., Marchetti N., Ferraro L., Regan R.F., Giunchedi P., Gavini E. (2015). Solid microparticles based on chitosan or methyl-beta-cyclodextrin: A first formulative approach to increase the nose-to-brain transport of deferoxamine mesylate. J. Control. Release.

[56] Akita T., Kimura R., Akaguma S., Nagai M., Nakao Y., Tsugane M., Suzuki H., Oka J.-I., Yamashita C. (2021). Usefulness of cell-penetrating peptides and penetration accelerating sequence for nose-to-brain delivery of glucagon-like peptide-2. J. Control. Release.

[57] Ozsoy Y., Güngör S. (2011). Nasal route: An alternative approach for antiemetic drug delivery. Expert Opin. Drug Deliv..

[58] Espinoza L.C., Silva-Abreu M., Clares B., Rodriguez-Lagunas M.J., Halbaut L., Canas M.A., Calpena A.C. (2019). Formulation dtrategies to improve nose-to-brain delivery of donepezil. Pharmaceutics.

[59] Liu L., Tian C., Dong B., Xia M., Cai Y., Hu R., Chu X. (2021). Models to evaluate the barrier properties of mucus during drug diffusion. Int. J. Pharm..

[60] Wu H., Hu K., Jiang X. (2008). From nose to brain: Understanding transport capacity and transport rate of drugs. Expert Opin. Drug Deliv..

[61] Graff C.L., Pollack G.M. (2005). Nasal Drug Administration: Potential for Targeted Central Nervous System Delivery. J. Curr. Chem. Pharm. Sci..

[62] Shingaki T., Hidalgo I.J., Furubayashi T., Sakane T., Katsumi H., Yamamoto A., Yamashita S. (2011). Nasal Delivery of P-gp Substrates to the Brain through the Nose–Brain Pathway. Drug Metab. Pharmacokinet..

[63] Bourganis V., Kammona O., Alexopoulos A., Kiparissides C. (2018). Recent advances in carrier mediated nose-to-brain delivery of pharmaceutics. Eur. J. Pharm. Biopharm..

[64] Dhuria S.V., Hanson L.R., Frey W.H., Ii W.H.F. (2008). Novel Vasoconstrictor Formulation to Enhance Intranasal Targeting of Neuropeptide Therapeutics to the Central Nervous System. J. Pharmacol. Exp. Ther..

[65] Pires A., Fortuna A., Alves G., Falcão A. (2009). Intranasal drug delivery: How, why and what for?. J. Pharm. Pharm. Sci..

[66] Perez-Caballero L., Torres-Sanchez S., Bravo L., Mico J.A., Berrocoso E. (2014). Fluoxetine: A case history of its discovery and preclinical development. Expert Opin. Drug Discov..

[67] Suwała J., Machowska M., Wiela-Hojeńska A. (2019). Venlafaxine pharmacogenetics: A comprehensive review. Pharmacogenomics.

[68] Patel R.G. (2017). Nasal Anatomy and Function. Facial Plast. Surg..

[69] Kumar A., Pandey A.N., Jain S.K. (2014). Nasal-nanotechnology: Revolution for efficient therapeutics delivery. Drug Deliv..

[70] Costa C.P., Moreira J., Amaral M.H., Lobo J.M.S., Silva A. (2019). Nose-to-brain delivery of lipid-based nanosystems for epileptic seizures and anxiety crisis. J. Control. Release.

[71] Quintana D.S., Westlye L.T., Rustan G.Ø., Tesli N., Poppy C.L., Smevik H., Tesli M., Røine M., Mahmoud R.A., Smerud K.T. (2015). Low-dose oxytocin delivered intranasally with Breath Powered device affects social-cognitive behavior: A randomized four-way crossover trial with nasal cavity dimension assessment. Transl. Psychiatry.

[72] Mittal D., Ali A., Md S., Baboota S., Sahni J.K., Ali J. (2013). Insights into direct nose to brain delivery: Current status and future perspective. Drug Deliv..

[73] Djupesland P.G. (2012). Nasal drug delivery devices: Characteristics and performance in a clinical perspective—A review. Drug Deliv. Transl. Res..

[74] Tong X., Dong J., Shang Y., Inthavong K., Tu J. (2016). Effects of nasal drug delivery device and its orientation on sprayed particle deposition in a realistic human nasal cavity. Comput. Biol. Med..

[75] Warnken Z.N., Smyth H.D., Watts A.B., Weitman S., Kuhn J.G., Williams R.O. (2016). Formulation and device design to increase nose to brain drug delivery. J. Drug Deliv. Sci. Technol..

[76] Chen Y., Liu Y., Xie J., Zheng Q., Yue P., Chen L., Hu P., Yang M. (2020). Nose-to-Brain Delivery by Nanosuspensions-Based in situ Gel for Breviscapine. Int. J. Nanomed..

[77] Cunha S., Forbes B., Lobo J.M.S., Silva A.C. (2021). Improving drug delivery for alzheimer's disease through nose-to-brain delivery using nanoemulsions, nanostructured lipid carriers (NLC) and in situ hydrogels. Int. J. Nanomed..

[78] Agrawal M., Saraf S., Saraf S., Dubey S.K., Puri A., Gupta U., Kesharwani P., Ravichandiran V., Kumar P., Naidu V. (2020). Stimuli-responsive In situ gelling system for nose-to-brain drug delivery. J. Control. Release.

[79] Zahir-Jouzdani F., Wolf J.D., Atyabi F., Bernkop-Schnürch A. (2018). In situ gelling and mucoadhesive polymers: Why do they need each other?. Expert Opin. Drug Deliv..

[80] Kanwar N., Sinha V.R. (2019). In Situ Forming Depot as Sustained-Release Drug Delivery Systems. Crit. Rev. Ther. Drug Carr. Syst..

[81] Mir M., Ahmed N., Rehman A.U. (2017). Recent applications of PLGA based nanostructures in drug delivery. Colloids Surf. B Biointerfaces.

[82] Kapoor D.N., Bhatia A., Kaur R., Sharma R., Kaur G., Dhawan S. (2015). PLGA: A unique polymer for drug delivery. Ther. Deliv..

[83] Desai K.G. (2016). Chitosan Nanoparticles Prepared by Ionotropic Gelation: An Overview of Recent Advances. Crit. Rev. Ther. Drug Carr. Syst..

[84] Prabaharan M. (2015). Chitosan-based nanoparticles for tumor-targeted drug delivery. Int. J. Biol. Macromol..

[85] Mohebbi S., Nezhad M.N., Zarrintaj P., Jafari S.H., Gholizadeh S.S., Saeb M.R., Mozafari M. (2019). Chitosan in Biomedical Engineering: A Critical Review. Curr. Stem Cell Res. Ther..

[86] Jana S., Sen K.K., Gandhi A. (2016). Alginate Based Nanocarriers for Drug Delivery Applications. Curr. Pharm. Des..

[87] Tønnesen H.H., Karlsen J. (2002). Alginate in Drug Delivery Systems. Drug Dev. Ind. Pharm..

[88] Severino P., Da Silva C.F., Andrade L.N., de Lima Oliveira D., Campos J., Souto E.B. (2019). Alginate Nanoparticles for Drug Delivery and Targeting. Curr. Pharm. Des..

[89] Thai H., Nguyen C.T., Thach L.T., Tran M.T., Mai H.D., Nguyen T.T.T., Le G.D., Van Can M., Tran L.D., Bach G.L. (2020). Characterization of chitosan/alginate/lovastatin nanoparticles and investigation of their toxic effects in vitro and in vivo. Sci. Rep..

[90] Reig-Vano B., Tylkowski B., Montané X., Giamberini M. (2020). Alginate-based hydrogels for cancer therapy and research. Int. J. Biol. Macromol..

[91] Ahmad E., Feng Y., Qi J., Fan W., Ma Y., He H., Xia F., Dong X., Zhao W., Lu Y. (2016). Evidence of nose-to-brain delivery of nanoemulsions: Cargoes but not vehicles. Nanoscale.

[92] Bahadur S., Pardhi D.M., Rautio J., Rosenholm J.M., Pathak K. (2020). Intranasal Nanoemulsions for Direct Nose-to-Brain Delivery of Actives for CNS Disorders. Pharmaceutics.

[93] Bonferoni M.C., Rossi S., Sandri G., Ferrari F., Gavini E., Rassu G., Giunchedi P. (2019). Nanoemulsions for “nose-to-brain” drug delivery. Pharmaceutics.

[94] Rinaldi F., Oliva A., Sabatino M., Imbriano A., Hanieh P.N., Garzoli S., Mastroianni C.M., De Angelis M., Miele M.C., Arnaut M. (2020). Antimicrobial Essential Oil Formulation: Chitosan Coated Nanoemulsions for Nose to Brain Delivery. Pharmaceutics.

[95] Pandey V., Kohli S. (2018). Lipids and Surfactants: The Inside Story of Lipid-Based Drug Delivery Systems. Crit. Rev. Ther. Drug Carr. Syst..

[96] Urquhart A.J., Eriksen A.Z. (2019). Recent developments in liposomal drug delivery systems for the treatment of retinal diseases. Drug Discov. Today.

[97] Pattni B.S., Chupin V.V., Torchilin V.P. (2015). New Developments in Liposomal Drug Delivery. Chem. Rev..

[98] Natsheh H., Touitou E. (2018). Phospholipid Magnesome—A nasal vesicular carrier for delivery of drugs to brain. Drug Deliv. Transl. Res..

[99] Natsheh H., Touitou E. (2020). Phospholipid Vesicles for Dermal/Transdermal and Nasal Administration of Active Molecules: The Effect of Surfactants and Alcohols on the Fluidity of Their Lipid Bilayers and Penetration Enhancement Properties. Molecules.

[100] Touitou E., Duchi S., Natsheh H. (2020). A new nanovesicular system for nasal drug administration. Int. J. Pharm..

[101] Tapeinos C., Battaglini M., Ciofani G. (2017). Advances in the design of solid lipid nanoparticles and nanostructured lipid carriers for targeting brain diseases. J. Control. Release.

[102] Garcês A., Amaral M.H., Lobo J.M.S., Silva A.C. (2018). Formulations based on solid lipid nanoparticles (SLN) and nanostructured lipid carriers (NLC) for cutaneous use: A review. Eur. J. Pharm. Sci..

[103] Czajkowska-Kośnik A., Szekalska M., Winnicka K. (2018). Nanostructured lipid carriers: A potential use for skin drug delivery systems. Pharmacol. Rep..

[104] Khosa A., Reddi S., Saha R.N. (2018). Nanostructured lipid carriers for site-specific drug delivery. Biomed. Pharmacother..

[105] Chen F., Jiang H., Xu J., Wang S., Meng D., Geng P., Dai D., Zhou Q., Zhou Y. (2020). In Vitro and In Vivo Rat Model Assessments of the Effects of Vonoprazan on the Pharmacokinetics of Venlafaxine. Drug Des. Dev. Ther..

[106] Schoretsanitis G., Haen E., Hiemke C., Endres K., Ridders F., Veselinovic T., Gründer G., Paulzen M. (2019). Pharmacokinetic correlates of venlafaxine: Associated adverse reactions. Eur. Arch. Psychiatry Clin. Neurosci..

[107] Cayero-Otero M.D., Gomes M.J., Martins C., Álvarez-Fuentes J., Fernández-Arévalo M., Sarmento B., Martín-Banderas L. (2019). In vivo biodistribution of venlafaxine-PLGA nanoparticles for brain delivery: Plain vs. functionalized nanoparticles. Expert Opin. Drug Deliv..

[108] Haque S., Md S., Fazil M., Kumar M., Sahni J.K., Ali J., Baboota S. (2012). Venlafaxine loaded chitosan NPs for brain targeting: Pharmacokinetic and pharmacodynamic evaluation. Carbohydr. Polym..

[109] Rizeq B.R., Younes N.N., Rasool K., Nasrallah G.K. (2019). Synthesis, Bioapplications, and Toxicity Evaluation of Chitosan-Based Nanoparticles. Int. J. Mol. Sci..

[110] Norman T.R., Olver J.S. (2021). Desvenlafaxine in the treatment of major depression: An updated overview. Expert Opin. Pharmacother..

[111] Tong G.-F., Qin N., Sun L.-W. (2016). Development and evaluation of Desvenlafaxine loaded PLGA-chitosan nanoparticles for brain delivery. Saudi Pharm. J..

[112] Ahmed S., Gull A., Aqil M., Ansari M.D., Sultana Y. (2019). Poloxamer-407 thickened lipid colloidal system of agomelatine for brain targeting: Characterization, brain pharmacokinetic study and behavioral study on Wistar rats. Colloids Surfaces B Biointerfaces.

[113] Fatouh A.M., Elshafeey A.H., Abdelbary A. (2017). Intranasal agomelatine solid lipid nanoparticles to enhance brain delivery: Formulation, optimization and in vivo pharmacokinetics. Drug Des. Dev. Ther..

[114] Fatouh A.M., Elshafeey A.H., Abdelbary A. (2017). Agomelatine-based in situ gels for brain targeting via the nasal route: Statistical optimization, in vitro, and in vivo evaluation. Drug Deliv..

[115] Jani P., Vanza J., Pandya N., Tandel H. (2019). Formulation of polymeric nanoparticles of antidepressant drug for intranasal delivery. Ther. Deliv..

[116] Alam M.I., Baboota S., Ahuja A., Ali M., Ali J., Sahni J.K. (2013). Intranasal infusion of nanostructured lipid carriers (NLC) containing CNS acting drug and estimation in brain and blood. Drug Deliv..

[117] Alam M.I., Baboota S., Ahuja A., Ali M., Ali J., Sahni J.K., Bhatnagar A. (2014). Pharmacoscintigraphic evaluation of potential of lipid nanocarriers for nose-to-brain delivery of antidepressant drug. Int. J. Pharm..

[118] Elsenosy F.M., Abdelbary G.A., Elshafeey A.H., Elsayed I., Fares A.R. (2020). Brain Targeting of Duloxetine HCL via Intranasal Delivery of Loaded Cubosomal Gel: In vitro Characterization, ex vivo Permeation, and in vivo Biodistribution Studies. Int. J. Nanomed..

[119] Khatoon M., Sohail M.F., Shahnaz G., Rehman F.U., Din F.U., Rehman A.U., Ullah N., Amin U., Khan G.M., Shah K.U. (2019). Development and Evaluation of Optimized Thiolated Chitosan Proniosomal Gel Containing Duloxetine for Intranasal Delivery. AAPS PharmSciTech.

[120] Purgato M., Papola D., Gastaldon C., Trespidi C., Magni L.R., Rizzo C., Furukawa T.A., Watanabe N., Cipriani A., Barbui C. (2014). Paroxetine versus other anti-depressive agents for depression. Cochrane Database Syst. Rev..

[121] Pandey Y.R., Kumar S., Gupta B.K., Ali J., Baboota S. (2015). Intranasal delivery of paroxetine nanoemulsion via the olfactory region for the management of depression: Formulation, behavioural and biochemical estimation. Nanotechnology.

[122] Silva S., Bicker J., Fonseca C., Ferreira N.R., Vitorino C., Alves G., Falcão A., Fortuna A. (2021). Encapsulated Escitalopram and Paroxetine Intranasal Co-Administration: In Vitro/In Vivo Evaluation. Front. Pharmacol..

[123] Motaleb M.A., Ibrahim I.T., Sayyed M.E., Awad G.A.S. (2017). (131)I-trazodone: Preparation, quality control and in vivo biodistribution study by intranasal and intravenous routes as a hopeful brain imaging radiopharmaceutical. Rev. Esp. Med. Nucl. Imagen Mol..

[124] Boche M., Pokharkar V. (2016). Quetiapine Nanoemulsion for Intranasal Drug Delivery: Evaluation of Brain-Targeting Efficiency. AAPS PharmSciTech.

[125] Shah B., Khunt D., Misra M., Padh H. (2016). Non-invasive intranasal delivery of quetiapine fumarate loaded microemulsion for brain targeting: Formulation, physicochemical and pharmacokinetic consideration. Eur. J. Pharm. Sci..

[126] Naik A., Nair H. (2014). Formulation and Evaluation of Thermosensitive Biogels for Nose to Brain Delivery of Doxepin. BioMed Res. Int..

[127] Eduardo T.Q., Angela A., Mateo L., Melanie L.Z., Valentina P.F., David C., Estefania C., Natalia R.S., Andrés V.C., Angel R.O. (2022). Ketamine for resistant depression: A scoping review. Actas Esp. Psiquiatr..

[128] Yavi M., Lee H., Henter I.D., Park L.T., Zarate C.A. (2022). Ketamine treatment for depression: A review. Discov. Ment. Health.

[129] Yao W., Cao Q., Luo S., He L., Yang C., Chen J., Qi Q., Hashimoto K., Zhang J.C. (2022). Microglial ERK-NRBP1-CREB-BDNF signaling in sustained antidepressant actions of (R)-ketamine. Mol. Psychiatry.

[130] Buddenberg T.E., Topic B., Mahlberg E.D., Silva M.A.D.S., Huston J.P., Mattern C. (2008). Behavioral Actions of Intranasal Application of Dopamine: Effects on Forced Swimming, Elevated Plus-Maze and Open Field Parameters. Neuropsychobiology.

[131] Chemuturi N.V., Donovan M.D. (2006). Metabolism of Dopamine by the Nasal Mucosa. J. Pharm. Sci..

[132] Brown V., Liu F. (2014). Intranasal Delivery of a Peptide with Antidepressant-Like Effect. Neuropsychopharmacology.

[133] Zangani C., Giordano B., Stein H., Bonora S., D’Agostino A., Ostinelli E.G. (2021). Efficacy of amisulpride for depressive symptoms in individuals with mental disorders: A systematic review and meta-analysis. Hum. Psychopharmacol. Clin. Exp..

[134] Hopkins S.C., Wilkinson S., Corriveau T.J., Nishikawa H., Nakamichi K., Loebel A., Koblan K.S. (2021). Discovery of Nonracemic Amisulpride to Maximize Benefit/Risk of 5-HT7 and D2 Receptor Antagonism for the Treatment of Mood Disorders. Clin. Pharmacol. Ther..

[135] Kishimoto T., Hagi K., Kurokawa S., Kane J.M., Correll C.U. (2022). Efficacy and safety/tolerability of antipsychotics in the treatment of adult patients with major depressive disorder: A systematic review and meta-analysis. Psychol. Med..

[136] Yuan B., Yuan M. (2022). Changes of Mental State and Serum Prolactin Levels in Patients with Schizophrenia and Depression after Receiving the Combination Therapy of Amisulpride and Chloroprothixol Tablets. Comput. Math. Methods Med..

[137] Gadhave D.G., Tagalpallewar A.A., Kokare C.R. (2019). Agranulocytosis-Protective Olanzapine-Loaded Nanostructured Lipid Carriers Engineered for CNS Delivery: Optimization and Hematological Toxicity Studies. AAPS PharmSciTech.

[138] El Assasy A.E.I., Younes N.F., Makhlouf A.I.A. (2019). Enhanced oral absorption of amisulpride via a nanostructured lipid carrier-based capsules: Development, optimization applying the desirability function approach and in vivo pharmacokinetic study. AAPS PharmSciTech.

[139] Gadhave D., Tupe S., Tagalpallewar A., Gorain B., Choudhury H., Kokare C. (2021). Nose-to-brain delivery of amisulpride-loaded lipid-based poloxamer-gellan gum nanoemulgel: In vitro and in vivo pharmacological studies. Int. J. Pharm..

[140] Lenze E.J., Mulsant B.H., Blumberger D.M., Karp J.F., Newcomer J.W., Anderson S., Dew M.A., Butters M.A., Stack J.A., Begley A.E. (2015). Efficacy, safety, and tolerability of augmentation pharmacotherapy with aripiprazole for treatment-resistant depression in late life: A randomised, double-blind, placebo-controlled trial. Lancet.

[141] Nelson J.C., Papakostas G.I. (2009). Atypical Antipsychotic Augmentation in Major Depressive Disorder: A Meta-Analysis of Placebo-Controlled Randomized Trials. Am. J. Psychiatry.

[142] Kirschbaum K.M., Uhr M., Holthoewer D., Namendorf C., Pietrzik C., Hiemke C., Schmitt U. (2010). Pharmacokinetics of acute and sub-chronic aripiprazole in P-glycoprotein deficient mice. Neuropharmacology.

[143] Piazzini V., Landucci E., Urru M., Chiarugi A., Pellegrini-Giampietro D.E., Bilia A.R., Bergonzi M.C. (2020). Enhanced dissolution, permeation and oral bioavailability of aripiprazole mixed micelles: In vitro and in vivo evaluation. Int. J. Pharm..

[144] Kumbhar S.A., Kokare C.R., Shrivastava B., Gorain B., Choudhury H. (2021). Antipsychotic Potential and Safety Profile of TPGS-Based Mucoadhesive Aripiprazole Nanoemulsion: Development and Optimization for Nose-To-Brain Delivery. J. Pharm. Sci..

[145] Sawant K., Pandey A., Patel S. (2016). Aripiprazole loaded poly(caprolactone) nanoparticles: Optimization and in vivo pharmacokinetics. Mater. Sci. Eng. C Mater. Biol. Appl..

[146] Taymouri S., Shahnamnia S., Mesripour A., Varshosaz J. (2021). In vitro and in vivo evaluation of an ionic sensitive in situ gel containing nanotransfersomes for aripiprazole nasal delivery. Pharm. Dev. Technol..

[147] Singh D.P., Rashid M., Hallan S., Mehra N.K., Prakash A., Mishra N. (2015). Pharmacological evaluation of nasal delivery of selegiline hydrochloride-loaded thiolated chitosan nanoparticles for the treatment of depression. Artif. Cells Nanomed. Biotechnol..

[148] Sridhar V., Gaud R., Bajaj A., Wairkar S. (2018). Pharmacokinetics and pharmacodynamics of intranasally administered selegiline nanoparticles with improved brain delivery in Parkinson's disease. Nanomedicine.

[149] Sridhar V., Wairkar S., Gaud R., Bajaj A., Meshram P. (2018). Brain targeted delivery of mucoadhesive thermosensitive nasal gel of selegiline hydrochloride for treatment of Parkinson's disease. J. Drug Target.

[150] Rukmangathen R., Yallamalli I.M., Yalavarthi P.R. (2019). Biopharmaceutical potential of selegiline loaded chitosan nanoparticles in the management of parkinson's disease. Curr. Drug Discov. Technol..

[151] Kumar S., Ali J., Baboota S. (2016). Design Expert^®^ supported optimization and predictive analysis of selegiline nanoemulsion via the olfactory region with enhanced behavioural performance in Parkinson’s disease. Nanotechnology.

[152] Kumar S., Dang S., Nigam K., Ali J., Baboota S. (2018). Selegiline nanoformulation in attenuation of oxidative stress and upregulation of dopamine in the brain for the treatment of parkinson's disease. Rejuvenation Res..

[153] Mishra N., Sharma S., Deshmukh R., Kumar A., Sharma R. (2019). Development and Characterization of Nasal Delivery of Selegiline Hydrochloride Loaded Nanolipid Carriers for the Management of Parkinson’s Disease. Central Nerv. Syst. Agents Med. Chem..

[154] Kaur P., Garg T., Vaidya B., Prakash A., Rath G., Goyal A.K. (2015). Brain delivery of intranasal in situ gel of nanoparticulated polymeric carriers containing antidepressant drug: Behavioral and biochemical assessment. J. Drug Target.

[155] Woo Y.S., Lim H.K., Wang S.-M., Bahk W.-M. (2020). Title Clinical Evidence of Antidepressant Effects of Insulin and Anti-Hyperglycemic Agents and Implications for the Pathophysiology of Depression—A Literature Review. Int. J. Mol. Sci..

[156] Hamer J.A., Testani D., Mansur R.B., Lee Y., Subramaniapillai M., McIntyre R.S. (2019). Brain insulin resistance: A treatment target for cognitive impairment and anhedonia in depression. Exp. Neurol..

[157] Zou X.H., Sun L.H., Yang W., Li B.J., Cui R.J. (2020). Potential role of insulin on the pathogenesis of depression. Cell Prolif..

[158] Nedelcovych M.T., Gadiano A.J., Wu Y., Manning A.A., Thomas A.G., Khuder S.S., Yoo S.-W., Xu J., McArthur J.C., Haughey N.J. (2017). Pharmacokinetics of Intranasal versus Subcutaneous Insulin in the Mouse. ACS Chem. Neurosci..

[159] Ren G., Xue P., Wu B., Yang F., Wu X. (2021). Intranasal treatment of lixisenatide attenuated emotional and olfactory symptoms via CREB-mediated adult neurogenesis in mouse depression model. Aging.

[160] Sasaki-Hamada S., Nakamura R., Nakao Y., Akimoto T., Sanai E., Nagai M., Horiguchi M., Yamashita C., Oka J.-I. (2017). Antidepressant-like effects exerted by the intranasal administration of a glucagon-like peptide-2 derivative containing cell-penetrating peptides and a penetration-accelerating sequence in mice. Peptides.

[161] Nakao Y., Horiguchi M., Nakamura R., Sasaki-Hamada S., Ozawa C., Funane T., Ozawa R., Oka J.-I., Yamashita C. (2016). LARETH-25 and β-CD improve central transitivity and central pharmacological effect of the GLP-2 peptide. Int. J. Pharm..

[162] Dickens M.J., Pawluski J.L. (2018). The HPA Axis During the Perinatal Period: Implications for Perinatal Depression. Endocrinology.

[163] Riem M., Kunst L., Bekker M., Fallon M., Kupper N. (2019). Intranasal oxytocin enhances stress-protective effects of social support in women with negative childhood experiences during a virtual Trier Social Stress Test. Psychoneuroendocrinology.

[164] Yoon S., Kim Y.-K. (2022). Possible oxytocin-related biomarkers in anxiety and mood disorders. Prog. Neuro-Psychopharmacol. Biol. Psychiatry.

[165] Chen Q., Zhuang J., Zuo R., Zheng H., Dang J., Wang Z. (2022). Exploring associations between postpartum depression and oxytocin levels in cerebrospinal fluid, plasma and saliva. J. Affect. Disord..

[166] Ross H., Cole C., Smith Y., Neumann I., Landgraf R., Murphy A., Young L. (2009). Characterization of the oxytocin system regulating affiliative behavior in female prairie voles. Neuroscience.

[167] Zhao J.L., Jiang W.T., Wang X., Cai Z.D., Liu Z.H., Liu G.R. (2020). Exercise, brain plasticity, and depression. CNS Neurosci. Ther..

[168] Castrén E., Monteggia L.M. (2021). Brain-Derived Neurotrophic Factor Signaling in Depression and Antidepressant Action. Biol. Psychiatry.

[169] Ma X.-C., Liu P., Zhang X.-L., Jiang W.-H., Jia M., Wang C.-X., Dong Y.-Y., Dang Y.-H., Gao C.-G. (2016). Intranasal Delivery of Recombinant AAV Containing BDNF Fused with HA2TAT: A Potential Promising Therapy Strategy for Major Depressive Disorder. Sci. Rep..

[170] Chen C., Dong Y., Liu F., Gao C., Ji C., Dang Y., Ma X., Liu Y. (2020). A Study of Antidepressant Effect and Mechanism on Intranasal Delivery of BDNF-HA2TAT/AAV to Rats with Post-Stroke Depression. Neuropsychiatr. Dis. Treat..

[171] Liu F., Liu Y.-P., Lei G., Liu P., Chu Z., Gao C.-G., Dang Y.-H. (2016). Antidepressant effect of recombinant NT4-NAP/AAV on social isolated mice through intranasal route. Oncotarget.

[172] Ma X.-C., Chu Z., Zhang X.-L., Jiang W.-H., Jia M., Dang Y.-H., Gao C.-G. (2016). Intranasal Delivery of Recombinant NT4-NAP/AAV Exerts Potential Antidepressant Effect. Neurochem. Res..

[173] Jiang J., Peng Y., Liang X., Li S., Chang X., Li L., Chang M. (2018). Centrally administered cortistation-14 induces antidepressant-like effects in mice via mediating ghrelin and GABA(A) receptor signaling pathway. Front. Pharmacol..

[174] Serova L., Laukova M., Alaluf L., Pucillo L., Sabban E. (2014). Intranasal neuropeptide Y reverses anxiety and depressive-like behavior impaired by single prolonged stress PTSD model. Eur. Neuropsychopharmacol..

[175] Oh J.-Y., Liu Q.F., Hua C., Jeong H.J., Jang J.-H., Jeon S., Park S.J.A.H.-J. (2020). Intranasal Administration of Melanin-Concentrating Hormone Reduces Stress-Induced Anxiety- and Depressive-Like Behaviors in Rodents. Exp. Neurobiol..

[176] Shi C.-G., Wang L.-M., Wu Y., Wang P., Gan Z.-J., Lin K., Jiang L.-X., Xu Z.-Q., Fan M. (2010). Intranasal Administration of Nerve Growth Factor Produces Antidepressant-Like Effects in Animals. Neurochem. Res..

[177] Beurel E., Toups M., Nemeroff C.B. (2020). The Bidirectional Relationship of Depression and Inflammation: Double Trouble. Neuron.

[178] Obermanns J., Krawczyk E., Juckel G., Emons B. (2021). Analysis of cytokine levels, T regulatory cells and serotonin content in patients with depression. Eur. J. Neurosci..

[179] Troubat R., Barone P., Leman S., Desmidt T., Cressant A., Atanasova B., Brizard B., El Hage W., Surget A., Belzung C. (2021). Neuroinflammation and depression: A review. Eur. J. Neurosci..

[180] Comai S., Melloni E., Lorenzi C., Bollettini I., Vai B., Zanardi R., Colombo C., Valtorta F., Benedetti F., Poletti S. (2021). Selective association of cytokine levels and kynurenine/tryptophan ratio with alterations in white matter microstructure in bipolar but not in unipolar depression. Eur. Neuropsychopharmacol..

[181] Rengasamy M., Brundin L., Griffo A., Panny B., Capan C., Forton C., Price R.B. (2021). Cytokine and Reward Circuitry Relationships in Treatment-Resistant Depression. Biol. Psychiatry Glob. Open Sci..

[182] Markova E.V., Shevela E.Y., Knyazeva M.A., Savkin I.V., Serenko E.V., Rashchupkin I.M., Amstislavskaya T.G., Ostanin A.A., Chernykh E.R. (2022). Effect of M2 Macrophage-Derived Soluble Factors on Behavioral Patterns and Cytokine Production in Various Brain Structures in Depression-Like Mice. Bull. Exp. Biol. Med..

[183] Wang Y.-L., Wang J.-X., Hu X.-X., Chen L., Qiu Z.-K., Zhao N., Yu Z.-D., Sun S.-Z., Xu Y.-Y., Guo Y. (2015). Antidepressant-like effects of albiflorin extracted from Radix paeoniae Alba. J. Ethnopharmacol..

[184] Zhao Z.-X., Fu J., Ma S.-R., Peng R., Yu J.-B., Cong L., Pan L.-B., Zhang Z.-G., Tian H., Che C.-T. (2018). Gut-brain axis metabolic pathway regulates antidepressant efficacy of albiflorin. Theranostics.

[185] Lindqvist D., Dhabhar F.S., James S.J., Hough C.M., Jain F.A., Bersani F.S., Reus V.I., Verhoeven J.E., Epel E.S., Mahan L. (2016). Oxidative stress, inflammation and treatment response in major depression. Psychoneuroendocrinology.

[186] Xu D., Qiao T., Wang Y., Wang Q.-S., Cui Y.-L. (2021). Alginate nanogels-based thermosensitive hydrogel to improve antidepressant-like effects of albiflorin via intranasal delivery. Drug Deliv..

[187] Zhu W.-Q., Wu H.-Y., Sun Z.-H., Guo Y., Ge T.-T., Li B.-J., Li X., Cui R.-J. (2022). Current Evidence and Future Directions of Berberine Intervention in Depression. Front. Pharmacol..

[188] Lee B., Shim I., Lee H., Hahm D.-H. (2018). Berberine alleviates symptoms of anxiety by enhancing dopamine expression in rats with post-traumatic stress disorder. Korean J. Physiol. Pharmacol..

[189] Fan J., Zhang K., Jin Y., Li B., Gao S., Zhu J., Cui R. (2018). Pharmacological effects of berberine on mood disorders. J. Cell. Mol. Med..

[190] Wang Q.-S., Li K., Gao L.-N., Zhang Y., Lin K.-M., Cui Y.-L. (2020). Intranasal delivery of berberine via in situ thermoresponsive hydrogels with non-invasive therapy exhibits better antidepressant-like effects. Biomater. Sci..

[191] Xu D., Qiu C., Wang Y., Qiao T., Cui Y.-L. (2021). Intranasal co-delivery of berberine and evodiamine by self-assembled thermosensitive in-situ hydrogels for improving depressive disorder. Int. J. Pharm..

[192] Zhang K., Lei N., Li M., Li J., Li C., Shen Y., Guo P., Xiong L., Xie Y. (2021). Cang-Ai Volatile Oil Ameliorates Depressive Behavior Induced by Chronic Stress Through IDO-Mediated Tryptophan Degradation Pathway. Front. Psychiatry.

[193] Chen B., Li J., Xie Y., Ming X., Li G., Wang J., Li M., Li X., Xiong L. (2019). Cang-ai volatile oil improves depressive-like behaviors and regulates DA and 5-HT metabolism in the brains of CUMS-induced rats. J. Ethnopharmacol..

[194] He C., Wang Z., Shi J. (2020). Pharmacological effects of icariin. Adv. Pharmacol..

[195] Xu S., Yu J., Zhan J., Yang L., Guo L., Xu Y. (2017). Pharmacokinetics, Tissue Distribution, and Metabolism Study of Icariin in Rat. BioMed Res. Int..

[196] Xu D., Lu Y.-R., Kou N., Hu M.-J., Wang Q.-S., Cui Y.-L. (2020). Intranasal delivery of icariin via a nanogel-thermoresponsive hydrogel compound system to improve its antidepressant-like activity. Int. J. Pharm..

[197] Hu W., Xie G., Zhou T., Tu J., Zhang J., Lin Z., Zhang H., Gao L. (2020). Intranasal administration of white tea alleviates the olfactory function deficit induced by chronic unpredictable mild stress. Pharm. Biol..

